# Synergistic effect of ginger extract in crosslinked polyvinyl alcohol and agar-agar: fabrication and *in vitro* study of 3D-printed scaffolds

**DOI:** 10.1039/d6ra06095a

**Published:** 2026-09-01

**Authors:** Rahila Batul, Rimsha Areej, Muhammad Sameet Ismat, Syed Muneeb Haider Gillani, Abdul Khaliq, Weaam Mohamed Khoj Ali, Hanan Abdelmawgoud Atia, Noreen Akhtar, Muhammad Atiq Ur Rehman

**Affiliations:** a College of Pharmacy, University of Hail Hail Saudi Arabia; b Department of Materials Science and Engineering, Ghulam Ishaq Khan Institute Topi Sawabi Pakistan; c Department of Materials Science and Engineering, Institute of Space Technology Islamabad Pakistan atique1.1@hotmail.com atiq.rehman@ist.edu.pk; d College of Engineering, University of Hail Hail Saudi Arabia; e College of Public Health, University of Hail Saudi Arabia

## Abstract

Effective wound management requires advanced biomaterials capable of providing bioactive functions that accelerate tissue regeneration. For this purpose, three-dimensional printed scaffolds were fabricated using biocompatible materials *via* the direct ink writing technique. The ink contained polyvinyl alcohol, agar-agar, and an ethanolic ginger extract at different ratios for enhanced viscosity and printability. The ginger extract was incorporated as a natural therapeutic agent due to its broad-spectrum antimicrobial and antioxidative properties. The manufactured scaffolds were crosslinked and lyophilized, followed by *in vitro* evaluations to examine their potential for wound healing applications. Crosslinking using calcium chloride (confirmed by Fourier transform infrared spectroscopy) helped in providing the interconnected porous structure. The porous structure facilitated hydrophilicity (∼3600%), yielding human dermal fibroblast cell viability, which was later confirmed through the water-soluble tetrazolium salt 8 assay. The disintegration of the 3D matrix in phosphate-buffered saline exhibited a controlled degradation rate (∼75% in 14 days), providing the burst and sustained release of 6-gingerol (∼79% in 14 days). The controlled release of 6-gingerol provided bactericidal efficacy against *Staphylococcus aureus* and *Escherichia coli*, as evidenced by turbidity tests. Collectively, these findings demonstrate that the 3D-printed scaffold (PVA/AA/Ginger) offers a promising bioactive solution due to their hydrophilicity, biocompatibility, and controlled drug release for soft-tissue engineering applications.

## Abstract 2

The graphical abstract presents the fabrication of 3D-printed scaffolds based on PVA/AA/Ginger using the DIW technique. Subsequently, the fabricated scaffolds undergoes to *in vitro* investigations (Designed using CanvaPro® (Canva, 2026)).

## Graphical abstract

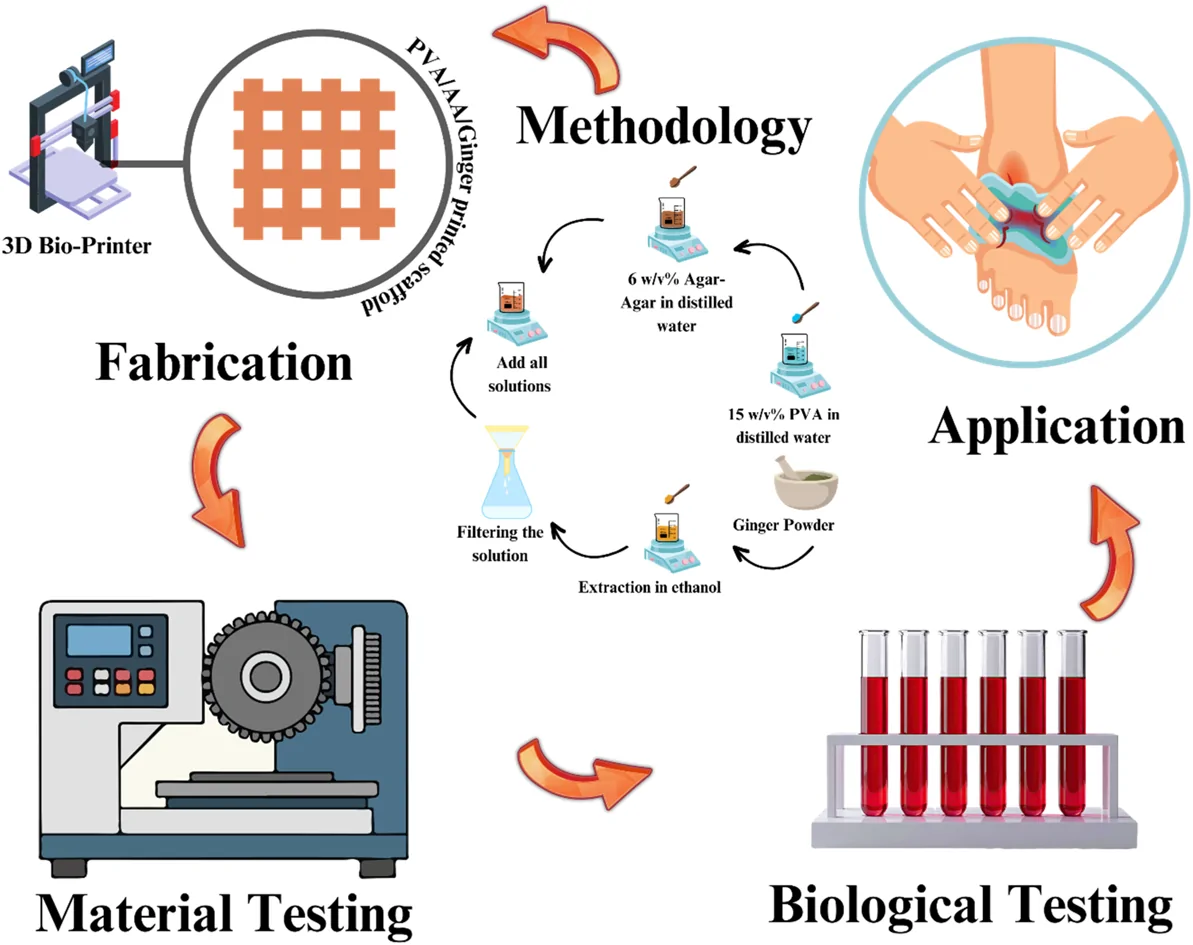

## 1. Introduction

The largest organ of the body (skin) contains water, protein, and minerals, which protect the internal tissue layers from external environmental attack.^1^ The skin comprises three distinct layers. The upper layer, known as the epidermis, protects the body from the outer environment. The middle layer, the dermis, consists primarily of collagen and provides structural strength. The innermost layer, the hypodermis, contains fat and connective tissues that insulate the body. Injuries such as burns, scars, scratches, or cuts can damage these layers, resulting in an increased risk for infection and impaired healing.^2^ Nowadays, wound infection is a leading cause of death worldwide. The latest report from the World Health Organization (WHO) stated that 3.1 million people died in 2021 due to injuries. About 90% of injury deaths happen in developing countries. The global wound care market is a multibillion-dollar industry, which was valued at around 23–25 billion USD in 2024 and is expected to reach 30–40 billion USD by 2030–2032.^3^ Wound healing is essential for reduce pain, pathogens, and infection and to promote recovery. Conventional wound dressings, such as gauze, lint, plasters, bandages, and cotton wool, are are widely used for wound management.^4^ However, upon removal, these dressings cause pain due to their poor bioactivity. To address this issue, modern dressing polymeric hydrogels have been introduced for wound repair applications.^5^ Hydrogels are soft and hydrophilic that can retain large amounts of water within their crosslinked structure, making them flexible, biocompatible, and useful for mimicking natural environments. Due to these properties, hydrogels are being effectively used in soft tissue regeneration. Ragab *et al.*^6^ synthesized a polyvinyl alcohol and chitosan-based hydrogel and investigated its wound repair efficiency, which exhibited antibacterial and regenerative properties through incorporated green-synthesized silver nanoparticles. Amini *et al.*^7^ developed hydrogels based on sodium alginate and gelatin, with the incorporation of selenium-doped deferoxamine-derived carbon quantum dots for wound closure. K. Zainab *et al.*^8^ prepared an injectable hydrogel based on silk sericin mixed with xanthan gum and examined its potential for skin burn applications. The hydrogels developed using different polymers are potent for soft tissue engineering, as they provide hydrophilicity, biocompatibility, and regenerative properties.

However, the irregular porosity in hydrogels is not suitable for deep wounds due to incongruous cell proliferation in pores, low strength and weak structural support.^9^ To counter these challenges, modern technology of three-dimensional-printed scaffolds have emerged for tissue engineering applications. Advanced additive manufacturing technologies, including fused deposition modeling (FDM), stereolithography (SLA), inkjet bioprinting, direct ink writing (DIW), selective laser sintering (SLS), laser-assisted bioprinting (LAB), and direct metal laser sintering (DMLS), are widely employed in tissue engineering applications.^10,11^ Polymeric materials, such as polylactic acid, are used in FDM as filaments, which melt within a heated extruder and print 3D scaffolds. The inkjet bioprinter employs a piezoelectric nozzle to deposit bioink droplets, achieving a high resolution. However, this technique is restricted to low-viscosity materials, which can lead to reduced cell viability as a result of elevated shear stress.^12,13^

DIW is an emerging technique to manufacture 3D structures using bioinks. The bioink solution of different biopolymers is directly extruded through a nozzle and forms a 3D structure. DIW is a direct extrusion-based additive manufacturing technique that builds 3D scaffolds by extruding bioinks through a needle in a layer-by-layer structure. The process relies on the ink's specialized rheological properties, which allow it to flow through a nozzle under shear stress and rapidly regain its solid-like state to maintain its shape upon deposition.^14,15^ These scaffolds provide essential mechanical strength and a biocompatible microenvironment that mimics the natural extracellular matrix (ECM) to support vital cellular activities. Ismat *et al.*^16^ fabricated a 3D-printed scaffold based on polyethylene oxide and locust bean gum using the DIW technique and reported the results of fabrication and *in vitro* characterizations for soft-tissue engineering applications. Gillani *et al.*^17^ developed a 3D-printed scaffold based on polyvinylpyrrolidone and carboxymethyl cellulose using the DIW method by incorporating collagen and oregano for wound-closure applications.

Natural polymers, such as alginate, chitosan, and guar gum, are very useful for the formation of biocompatible 3D-printed scaffolds.^18^ In this study, agar-agar (AA) is used to fabricate biocompatible 3D scaffolds. Agar-agar is a natural, jelly-like substance derived from red algae, widely used as a vegan thickener and gelling agent. Due to its biocompatibility, thermo-reversible gelling, high water retention, and tunable mechanical properties, it can be widely used in tissue-engineering applications.^19^ Polyvinyl alcohol (PVA) is a versatile, biocompatible polymer used in tissue engineering for scaffolds, hydrogels, and membranes due to its good mechanical strength, water retention, and FDA approval for some uses, especially in nerve and skin repair.^20^ Gillani *et al.*^21^ fabricated a 3D-printed scaffold using the DIW technology based on agar-agar and reported the regenerative properties of the 3D scaffold, which helped in wound closure. Shamloo *et al.*^22^ developed 3D-printed scaffolds based on PVA with gelatin and presented excellent compatibility and regeneration properties *via in vitro* and *in vivo* testing.

For wound repair, antibacterial efficacy is an essential requirement to kill bacteria at wound sites.^23^ Synthetic antibiotics exert the requisite antibacterial activity; however, elevated concentrations may induce toxic effects in certain instances.^24^ Plant-based natural herbs, such as *Aloe vera*, *curcumin*, and *calendula*, are likely to be used for their biological properties by incorporating them into the hydrogel and 3D scaffolds.^25^ Ginger contains a major constituent, [6]-gingerol, which shows promise in tissue engineering due to its potent anti-inflammatory, antioxidant, and cell-modulating properties, which help create a pro-regenerative microenvironment.^26,27^ Abdulzahra *et al.*^28^ reported the antibacterial activity of ginger extract against various pathogens and studied the comparative results with garlic bactericidal activity.

Herein, the authors introduce a novel solution for wound repair by mixing biocompatible ingredients, comprising PVA, AA, and ginger. The optimized ink was extruded through a DIW extruder to fabricate a biocompatible 3D-printed scaffold. Following this, the fabricated scaffold was examined through *in vitro* investigations (swelling, degradation, drug-release kinetics, and cell viability) to evaluate its potential for wound healing applications. The novelty lies in integrating ginger extract within a DIW-printable PVA/AA system capable of simultaneous structural support, controlled degradation, sustained gingerol release, antibacterial activity and fibroblast compatibility. The *in vivo* tests of the PVA/AA/Ginger scaffolds may explore more findings for soft tissue engineering applications, which may be investigated in the future.

## 2. Materials and methods

### 2.1. Materials

Agar-agar (AA) ([C_12_H_18_O_9_]_n_), polyvinyl alcohol (PVA) ([–CH_2_–CH(OH)–]_*n*_), phosphate-buffered saline (PBS), fetal bovine serum (FBS) and Dulbecco's Modified Eagle Medium (DMEM) were purchased from Sigma-Aldrich®. A Vascular Endothelial Growth Factor (VEGF) ELISA kit was purchased from FineTest, Wuhan, China. The Ginger powder was purchased from National Foods^©^, Islamabad, Pakistan.

### 2.2. Ink preparation

**Fig. 1:**
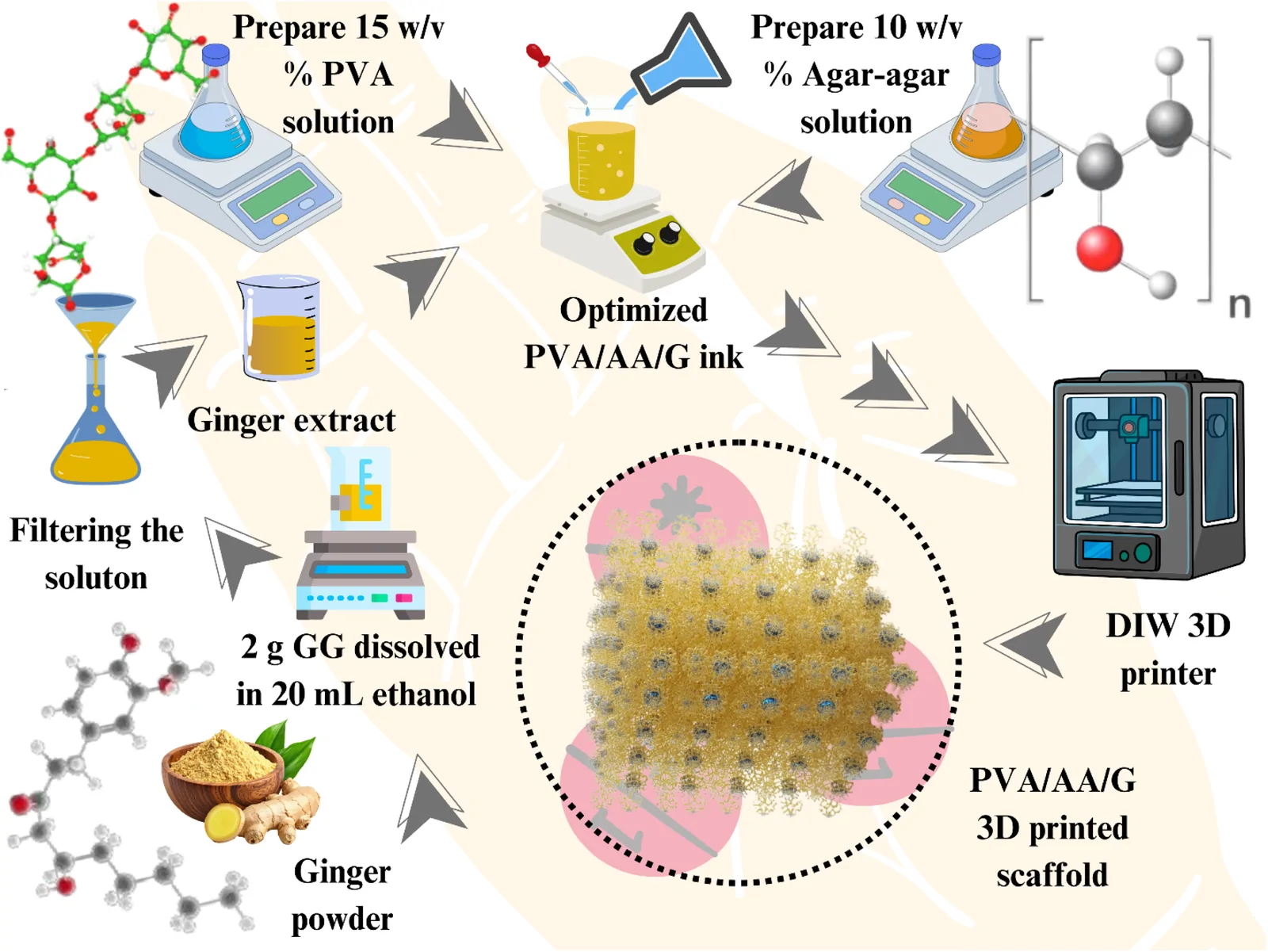
Schematic of the fabrication of the 3D-printed scaffolds *via* the DIW technique using biocompatible materials PVA and AA loaded with a natural herb ginger (Designed using CanvaPro (Canva, 2026)).

The ink of 15% w/v PVA and 10% w/v AA solutions was prepared in distilled water and stirred magnetically at 350 rpm and 80 °C. Thereafter, 2 g of ginger powder was dissolved in 20 mL of ethanol and stirred at 300 rpm for 30 min. AA and PVA were added in ratios of 50 : 50, 70 : 30, and 60 : 40; 70 : 30 was selected as the optimal ratio owing to the superior printability and viscosity offered at this ratio, as listed in Table 1. The extract was obtained by filtering the solution through a filter paper (Whatman No. 1). Based on our previous study,^16^ 50 µg/mL ginger extract was dissolved in the final solution of PVA/AA. Following this, the optimized solution was 3D printed using DIW (customized bioprinter) at optimized printing parameters (listed in Table 1). The schematic of the PVA/AA/Ginger 3D scaffolds is shown in Fig. 1. The optimization of the DoE was mainly aimed at finding an ink that showed reliable performance with respect to printing, filament formation, and structure retention. A thorough analysis of all formulations from a biological and mechanical perspective was not within the scope of this work's optimization phase. Accordingly, the optimized 30 : 70 ratio represented the best printable formulation, which was later tested to validate its functionality.

**Table 1:** DoE approach to achieve optimized ink solution based on PVA/AA/Ginger for the fabrication of 3D-printed scaffolds *via* the DIW technology (✗ represents irregular printing, ∅ represents the printing but in merged layers, and ✓ represents the optimum printing)

Sr No.	Concentration (W/V%)		Ratio		Results
	PVA	AA	PVA	AA	Printability
1	5	3	50	50	✗
			40	60	✗
			30	70	✗
	7	5	50	50	✗
			40	60	✗
			30	70	∅
2	10	7	50	50	✗
			40	60	✗
			30	70	∅
	15	10	50	50	✗
			40	60	✗
			30	70	✓

### 2.3. Rheology

The Brookfield DV-III rheometer was utilized to analyze the rheology of the ink. The flow properties of the prepared solution were identified. Viscosity and yield values were measured to determine the general flowability properties of the developed solution. The three interval thixotropy test (3iTT) was used to evaluate the ability of the solution to sustain changes in the shear rates.

### 2.4. Fabrication using DIW

The scaffold was designed using Computer-Aided Design (CAD). After that, the 3D design was converted into G-code using a Prusa Slicer® for 3D printing. The optimized ink was used to print the PVA/AA and PVA/AA/Ginger scaffolds using DIW, as discussed in Section 3.2. The structural stability of the scaffolds was achieved by crosslinking PVA with agar-agar. The fabricated scaffold was crosslinked by immersion in a CaCl_2_ bath and lyophilized.

### 2.5. Morphological analysis

The optical micrographs of the PVA/AA and PVA/AA scaffolds loaded with extracted ginger, taken *via* Carl Zeiss® using Extended Depth of Field (EDF), provided a clear, focused image of the entire surface topography of the scaffolds, even if the latter had varied heights and depths. Through these micrographs, detailed information about the surface morphology, such as pore size and pore uniformity, as well as surface properties, including roughness or smoothness, of the scaffolds was visible.^29^ Moreover, the pore size was measured using the ImageJ software. The morphological analysis of the scaffolds was performed by Field Emission Scanning Electron Microscope (FE-SEM, LEO 435 VP, MIRA TESCAN).

### 2.6. ATR-FTIR

Attenuated Total Reflection-Fourier Transform Infrared Spectroscopy (ATR-FTIR, Nicolet 6700, Thermo Scientific) was employed to identify the chemical groups in individual materials and the PVA/AA and PVA/AA/Ginger 3D scaffolds. The ATR-FTIR analysis of the samples was performed in the transmit mode in the range from 4000 cm^−1^ to 400 cm^−1^ with a resolution of 4 cm^−1^. Background was measured initially to remove the noise peaks. After that the sample placed and peaks were identified.

### 2.7. Swelling study

Swelling experiments were carried out to analyze the swelling property of the PVA/AA and PVA/AA/Ginger scaffolds. Three scaffold specimens, with dimensions of 4 mm × 4 mm × 1.5 mm, were weighed initially, placed inside a sample box, and submerged in a solution of 10 mL of distilled water. Following this, the scaffolds were removed from the water, and the excess water was wiped off, with the average swelling ratio calculated after 12 h by following eqn (1):1
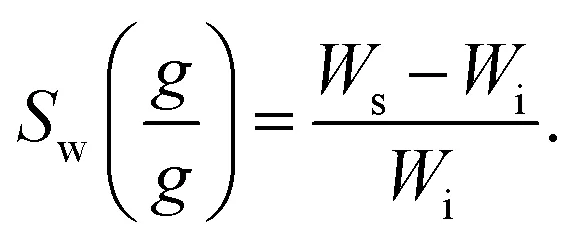
Here, *S*_w_ represents the swelling ratio, *W*_s_ represents the scaffold weight after swelling, and *W*_i_ shows the initial weight of the scaffold before swelling.

### 2.8. Mechanical study

The rectangular specimen scaffolds (15 × 10 × 1 mm^3^) underwent micro-tensile testing according to ASTM D882 standards using a Linkam TST350 testing platform fitted with a 200 N load cell. The test was carried out at ambient temperature (∼22.1 °C), with a constant crosshead speed of 5 mm min^−1^ up to breakage. Stress–strain curves were plotted using force and displacement data obtained during the process to calculate important mechanical values, such as ultimate tensile strength and fracture strain.

### 2.9. Degradation study

The degradation analysis using mass loss for the PVA/AA and PVA/AA/Ginger scaffolds was performed in PBS at 37 °C for 14 days. To start with, dried scaffolds with dimensions of 4 × 4 mm^2^ were carefully weighed, after which they were moved into 25 mL PBS solutions. The scaffolds were intermittently taken, and the mass loss was measured for 14 days. After each interval, the scaffolds were washed using distilled water, dried until they attained a constant weight, and reweighed. The percentage degradation was determined through eqn (2).

2 Mass loss (%) = ((*W*_i_ − *W*_s_)/*W*_i_) × 100

Here, *W*_s_ represents the scaffold's mass at a given time, and *W*_i_ denotes its initial dry mass.

### 2.10. Drug release

The release of the gingerol loaded in the scaffold was measured by immersing the scaffold in a 10 mL PBS solution placed in a shaking incubator. Drug release was measured at periodic intervals using a UV-visible spectrophotometer (Thermo Scientific, Genesys 10S), recording the optical density at 292 nm (OD_292_). The calibration curve of gingerol was drawn *via* absorbance at various concentrations. The absorbance value of gingerol was measured and compared to the calibration curve. Finally, the cumulative release of gingerol from the scaffold was determined for 14 days. The release study was conducted in triplicate, and average values and standard deviations were reported. Moreover, the drug-loading efficiency of the fabricated PVA/AA/Ginger scaffolds was determined by dissolving one scaffold in 50 mL of ethanol under continuous magnetic stirring until complete dissolution was achieved. The concentration of encapsulated ginger was quantified using a UV-visible spectrophotometer at OD_292_, employing a previously established calibration curve. The theoretical ginger concentration was calculated from the initial amount incorporated into the bioink (50 µg mL^−1^). The drug-loading efficiency was calculated using eqn (3)3
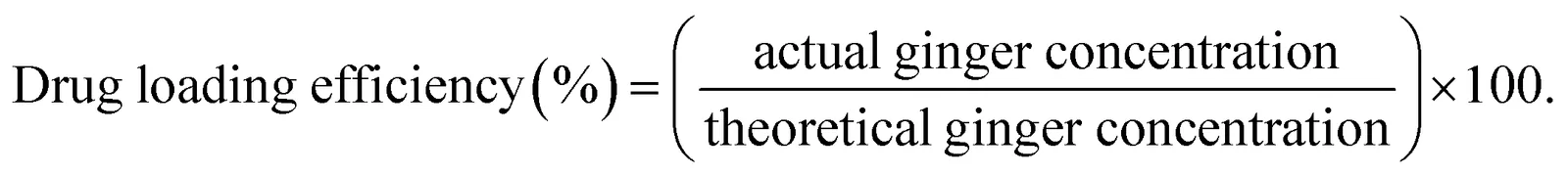


### 2.11. Antibacterial study

The turbidimetric study of bacterial growth in the liquid media with and without the loaded ginger in the PVA/AA scaffold was performed using the UV-vis Spectrophotometer. Overnight-grown *E. coli* and *S. aureus* were diluted to set their absorbance at 0.015 ± 0.002. The nutrient broth media (Oxoid – UK) autoclaved at 121 °C for 20 minutes. The absorbency of both bacterial was normalized to 0.015 ± 0.002 at OD_600_. After that, the samples were exposed to the prepared samples, with the bare samples acting as a control. After the incubation process for 24 h, the absorbency of the prepared samples was calculated again at the same wavelength. The absorbency of both the samples was analyzed with respect to the control sample, and the antibacterial property was evaluated.^30^

The disk diffusion was also conducted to evaluate the qualitative bactericidal efficacy of the PVA/AA/Ginger-based 3D scaffold. The scaffolds were evaluated against *Escherichia coli* (*E. coli*) and *Staphylococcus aureus* (*S. aureus*) bacteria. Nutrient Agar (Acquired from Sigma-Aldrich) was made under the specified conditions and autoclaved at 121 °C for 45 minutes. The melted agar was poured into sterile Petri dishes and allowed to solidify. The bacterial cultures of *E. coli* and *S. aureus* that had grown overnight were adjusted to an OD_600_ of 0.015 ± 0.002 and then spread evenly across the solidified agar plates with the help of a sterile glass spreader. The three samples, standard antibiotic (NC), PVA/AA (PC) and PVA/AA/Ginger (Sample), were then placed in the Petri dishes containing the bacteria and incubated in an incubator for 48 h at 37 °C. The inhibition zones in the agar culture plates were photographed after incubation, and their sizes were quantitatively determined through the ImageJ® software.^31^

### 2.12. Biocompatibility and VEGF release

The cytocompatibility of the PVA/AA and PVA/AA/Ginger scaffolds was evaluated by the WST-8 method using Human Dermal Fibroblast Cells (HDFa). This was done by sterilizing the scaffolds under UV light for 24 h. HDFa cells were cultured and seeding at a density of 1 × 10^5^ cells per well and incubated carefully at 37 °C under air and controlled humidity. The cytocompatibility was analyzed by adding the WST-8 reagent to the cell culture media on days 1, 3, 5, and 7.

An important aspect of our study was the use of the WST-8 reagent, which was reduced by live cells to yield formazan dye, which was soluble in water. This was quantified at OD_450_ nm using the microplate reader. A high absorbance value was linked to high cell viability. The difference in absorbance values between the PVA/AA and PVA/AA/Ginger scaffolds compared with TCP determine the cytocompatibility of the scaffolds, with high values showing cytocompatibility and cell survival, as explained above. Cell viability is determined based on eqn (4).4
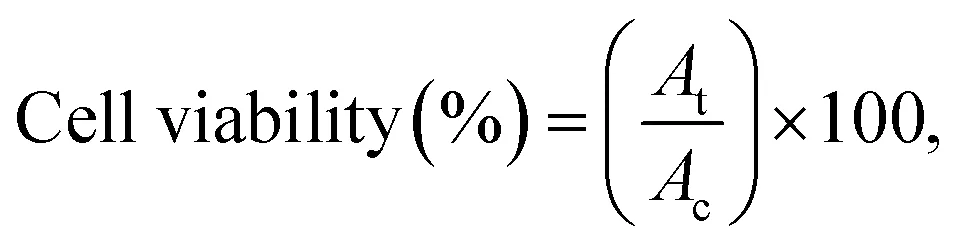
where *A*_t_ is the absorbance of the treated wells, and *A*_c_ represents the absorbance of the control wells.

ELISA assay (Human VEGF ELISA Kit; FineTest) was employed to assess the Vascular Endothelial Growth Factor (VEGF) release by human dermal fibroblasts (HDFa), as well as the PVA/AA/Ginger scaffolds, for 14 days. Fresh media were added periodically to ensure HDFa survival; however, this involved continuous culturing for 14 days. The sample's absorbance was retrieved at OD_450_ nm from each well and assessed in the ELISA microplate reader (Multiskan FC; Thermo Fisher).

### 2.13 *In silico* analysis

To validate the molecular basis of the biological activity of the scaffold, the *in silico* docking process was carried out utilizing [6]-gingerol (PubChem CID: 442438) against the following targets: Integrin αvβ3 (1L5G), DNA gyrase B (6RQU), VEGFR2 (4ASE) and COX-2 (5KIR). The docking process was performed using the AutoDock Vina 1.2.3 software through Google Colab®, with molecular preparation done through RDKit, OpenBabel and Meeko. The receptors were devoid of any water molecules and heteroatoms, while the grid boxes were placed at the active sites of each receptor in question. The docking procedure was performed with an exhaustiveness of 10, with the ranking criteria being the minimum binding affinity (kcal mol^−1^).

### 2.14. Statistical analysis

Statistical analysis of the experimental results was performed to assess the major variations between the values. Also, one-way analysis of variance (ANOVA) was performed to confirm the significant differences (*, *p* < 0.05) in the findings. Tukey's mean comparison technique was adopted to establish the statistics of the results. These processes were conducted using the OriginPro 8.5 software, and the results were presented in terms of mean and standard deviation for triplicate analysis. All the samples and the biological properties of the materials were analyzed in triplicate (*n* = 3).

## 3. Results and discussion

### 3.1. Rheological analysis

The rheological behavior of the bioinks plays a crucial role in 3D printing *via* DIW of hydrogels, as it determines the flowability and printability under the shear stresses offered by the DIW 3D printer. The polymeric blends that show a shear-thinning effect are considered preferable in extrusion-based 3D printing, as 3D printing requires the breaking of polymeric chains at specific shear stresses, after which the blends become flowable and undergo 3D printing.^32–34^ The rheological behavior of PVA and PVA/AA/Ginger is illustrated in Fig. 3. The flow curve of PVA is depicted in Fig. 3(A). The PVA solution showed a significant shear-thinning effect, as confirmed by the Herschel–Bulkley model for viscoplastic fluids, with the flow behavior index “*n*” of 0.63, which proved the flowability of PVA under shear stresses.^35^ The PVA solution exhibited high viscosity at low shear rates, while the viscosity values decreased with the increase in shear rates, confirming the pseudoplastic behavior of PVA. The increase in the values of shear stresses with the increase in shear rates confirmed the breakdown of the molecular chains of PVA at high rates and at the specific shear stress of 82 dyne cm^−2^; the slow increase in shear stress was observed, which confirmed the flowability after the mentioned shear stress. The thixotropic behavior of PVA is shown in Fig. 3(B). The solution exhibited high viscosity during the first interval at a shear rate was 6 s^−1^. When the shear rate was changed to 12 s^−1^ in the second interval, the viscosity values suddenly decreased. This was followed by a recovery in the viscosity in the third interval when the shear rate was changed back to 6 s^−1^. For the PVA solution, an 84% viscosity recovery rate was noted. The PVA/AA/Ginger blend's flow characteristics are illustrated in Fig. 3(C). In comparison to PVA, the blend of PVA/AA/Ginger demonstrated higher viscosities and shear stresses. The increased viscosity values were attributed to the addition of AA to PVA, which formed interconnections and 3D networking. The blend of PVA/AA/Ginger showed a relatively high shear-thinning effect in comparison to pure PVA, with a flow behavior index “*n*” value of 0.52, which confirmed the improved flowability under shear rates. The values of shear stress increased significantly due to the addition of AA in PVA, which was attributed to the bonding between PVA and AA and the high viscosity of AA. The increased shear stresses were attributed to the high shear rates during flow, and the molecular chains of PVA and AA were expected to start breaking after 136 dyne cm^−2^ and promote flowability after the mentioned shear stress. The thixotropic behavior of the PVA/AA/Ginger blend is shown in Fig. 3(D). Similar to the thixotropic behavior of PVA, the sudden decrease in the viscosity of the PVA/AA/Ginger blend was examined in the second interval when the instant shear rate of the blend was 12 s^−1^. The sudden decrease in viscosity values was attributed to the shear-thinning behavior of the blend. The blend of PVA/AA/Ginger showed improved viscosity retention of 96% within 5 s in the third interval. The improved viscosity retention was attributed to the strong bonding and 3D networking between PVA and AA. Hence, the improved flowability and thixotropic behavior of the PVA/AA/Ginger blend make it an ideal candidate for extrusion-based 3D printing.

**Fig. 2:**
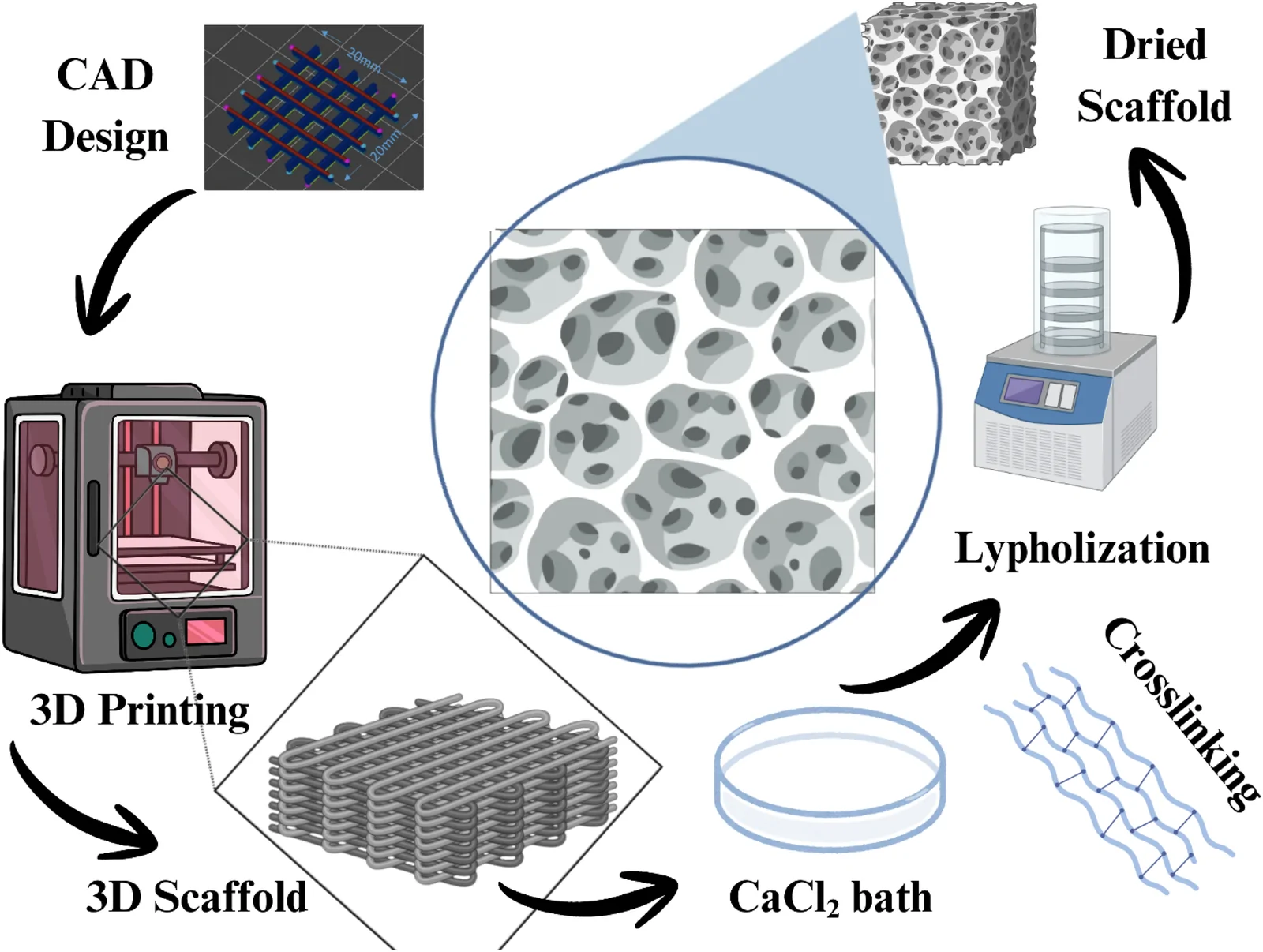
Schematic route of the 3D design model of the scaffold to printing, crosslinking and freeze-drying (Designed using CanvaPro (Canva, 2026)).

**Fig. 3:**
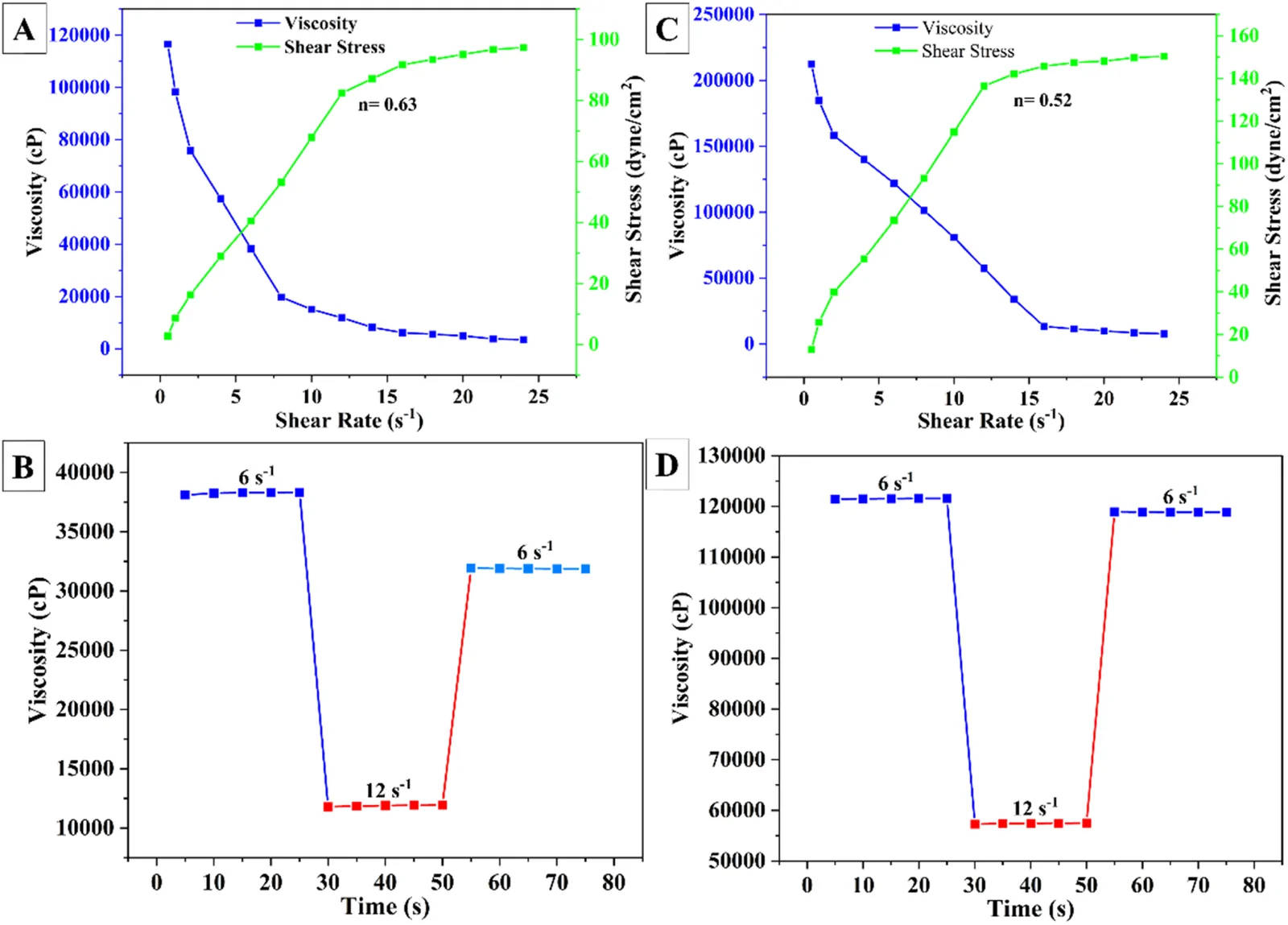
(A) Flow curve of PVA. (B) 3-interval thixotropic behavior of the PVA solution. (C) Flow curve of the PVA/AA/Ginger blend. (D) 3-interval thixotropic behavior of the PVA/AA/Ginger blend.

### 3.2. Fabrication using DIW

**Fig. 4:**
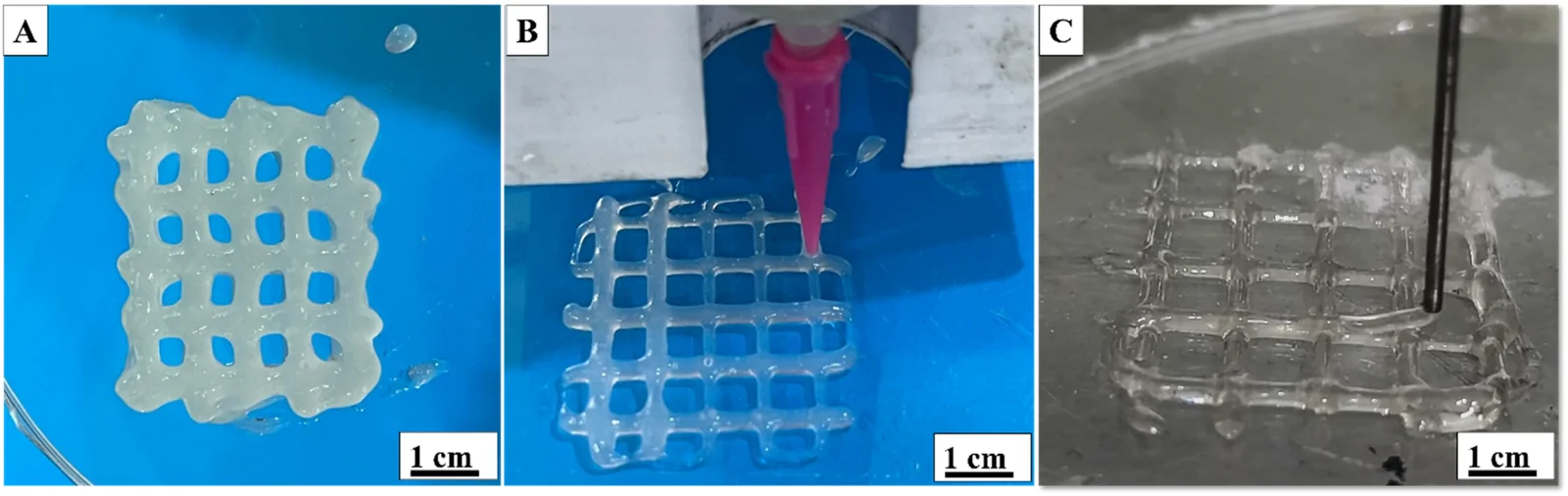
Printability of the PVA/AA/Ginger blend at different ratios (V/V). (A) 50 : 50 (PVA : AA), (B) 40 : 60 (PVA : AA), (C) 30 : 70 (PVA : AA).

The printability of bioinks plays a vital role in the fabrication of the scaffolds for wound-repair applications, as it impacts the structural support and fibroblast cell adhesion. Ideally, bioinks that exhibit homogeneous layer fidelity are preferred in wound-healing applications. The polymeric bioinks that show low viscosities and surface tension are not considered in extrusion-based 3D printing, as they cause the merging of printed layers, resulting in poor printability.^21,36,37^ The parameters adopted for the 3D printing of the PVA/AA/Ginger blend are listed in Table 2. The scaffolds were kept porous, with a fill density of 15%, to ensure the proper nutrient transport required in tissue-engineering applications. The fill angle was kept at 45° to achieve better printability. The flow speed and printing speed were adjusted to 16 mm^3^ s^−1^ and 3 mm s^−1^, respectively, due to the improved flowability at the shear rate mentioned in Section (3.1). The printability of different ratios of PVA and AA is demonstrated in Fig. 4. The blend of 50 : 50 (PVA : AA) showed poor printability, with an irregular structure and non-homogeneous layer fidelity, as illustrated in Fig. 4(A). The poor printability of the blend was attributed to the low viscosities and surface tension of PVA, which resulted in the merging of the printed layers. In comparison to the 50 : 50 (PVA : AA) blend, the blend of 40 : 60 (PVA : AA) showed better printability, as depicted in Fig. 4(B). The improved printability was due to the increased amount of AA in the ink blend solution, which increased the viscosity and surface tension of the blend. However, the merging of the printed layers was also observed for the 40 : 60 (PVA : AA) blend due to the low surface tension and viscosity of the blend. Suitable printability was observed for the 30 : 70 (PVA : AA) blend due to the increment in the AA concentration in the blend, as shown in Fig. 4(C). The increment in the AA concentration in the blend resulted in better printability, which was attributed to the thickening ability and high viscosity of AA, as required in DIW 3D printing. Moreover, the homogeneous layer fidelity was observed for the mentioned blend, which played a crucial role in fibroblast cell adhesion and migration. Hence, the blend of 30 : 70 (PVA : AA) proved suitable for DIW 3D printing (Table 1).

**Table 2:** Printing parameters adjusted to 3D print the PVA/AA/Ginger scaffold

Sr. No.	Printing parameters	Values
1	Fill density	15%
2	Fill angle	45°
3	Bed and nozzle temperature	25 °C
4	Fill pattern	Rectilinear
5	Flow speed	16 mm^3^ s^−1^
6	Nozzle inner diameter	540 µm
7	Number of layers	9
8	Printing speed	3 mm s^−1^
9	Layer height	0.33 mm

**Fig. 5:**
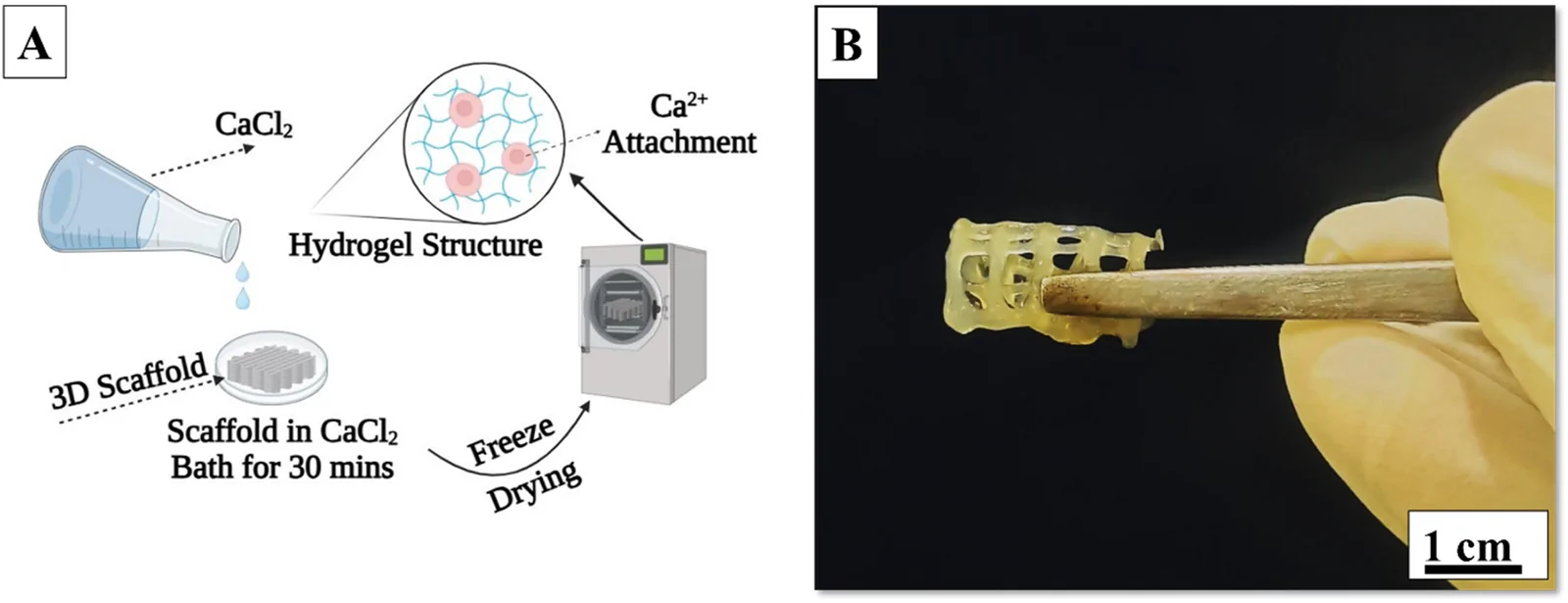
(A) Crosslinking mechanism of the 3D-printed PVA/AA/Ginger blend. (B) Bending ability of the scaffold after crosslinking with CaCl_2_ (Designed using CanvaPro (Canva, 2026)).

The collected blend of PVA/AA/Ginger was crosslinked using an ionic crosslinker, CaCl_2_. The blend was immersed in a CaCl_2_ bath for 30 min, allowing Ca^2+^ ions to diffuse into the matrix and interact primarily with the abundant, hydroxyl-rich polymer chains of both polyvinyl alcohol (PVA) and agar-agar (AA). Rather than the classical electrostatic crosslinking *via* the sparse carboxylate groups of AA, the divalent Ca^2+^ ions acted as coordination centers that bridged the neutral hydroxyl –OH groups of both polymers. The mechanism of crosslinking is illustrated in Fig. 5(A). In addition, the PVA/AA/Ginger hydrogel possessed bending ability due to the successful crosslinking and 3D networking, as shown in Fig. 5(B). The stability of the 3D-printed scaffolds after the CaCl_2_ treatment was governed by the formation of an ionically crosslinked network, which enhanced structural integrity and resistance to rapid dissolution in aqueous environments.^38^ The scaffolds retained their geometrical fidelity and porous architecture under physiological conditions, indicating sufficient mechanical coherence after crosslinking. This stability was further supported by their controlled degradation behavior over the study period, demonstrating that the network did not undergo premature disintegration.^39^ Additionally, the crosslinked structure contributed to maintaining porosity and swelling capacity, which were essential for fluid uptake and nutrient transport. Overall, the scaffolds exhibit a balanced stability profile, remaining intact during early-stage application while gradually degrading in a controlled manner, which is desirable for wound-healing applications.^40^

### 3.3. Morphological analysis

**Fig. 6:**
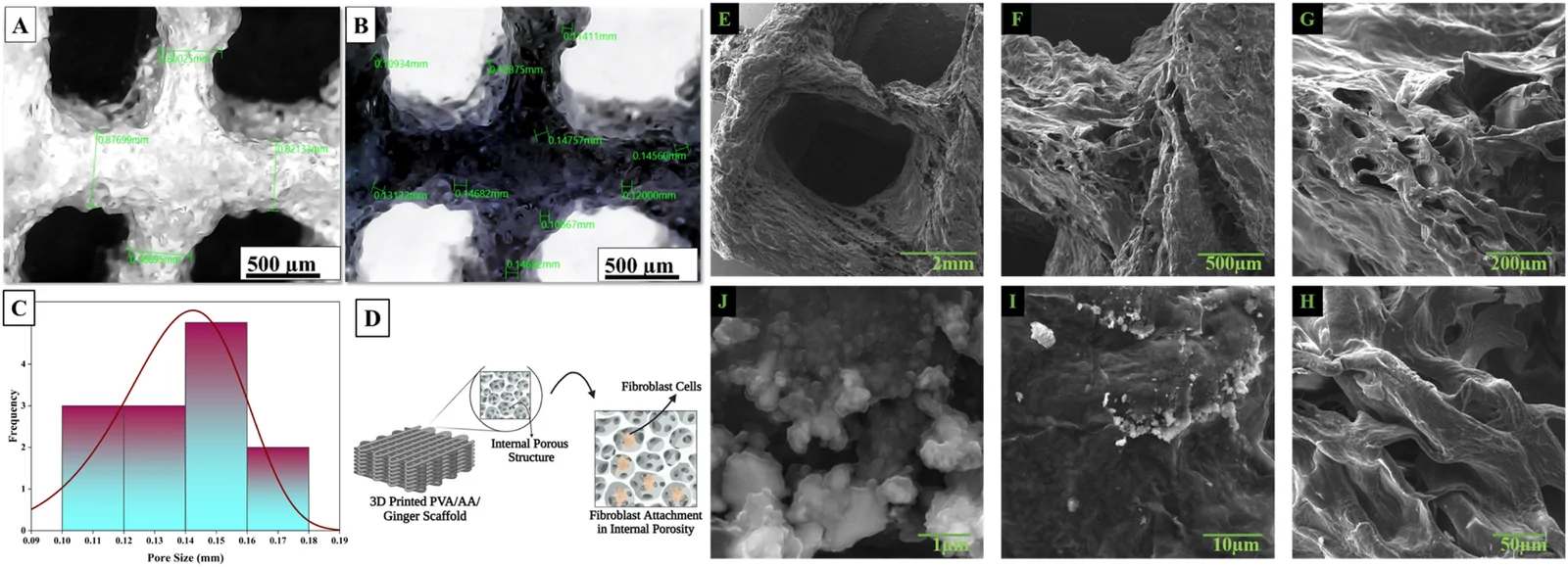
Microscopic images of the scaffolds. (A) Layer fidelity of the 3D-printed PVA/AA/Ginger scaffolds. (B) Internal porous structure of 3D-printed scaffolds. (C) Pore size (mm) measured through the ImageJ® software. (D) Mechanism of fibroblast adhesion in the internal porous structure of the 3D-printed scaffolds (Designed using CanvaPro (Canva, 2026)). (E–J) SEM images of the fabricated PVA/AA/Ginger scaffolds.

The structural morphology of the 3D-printed hydrogels is very vital in skin tissue engineering applications, as it yields fibroblast cell adhesion and proliferation on the surfaces of the scaffolds. The layer fidelity of the 3D-printed hydrogels should be homogeneous to support the structure of the hydrogels. The morphology of the 3D-printed PVA/AA/Ginger scaffolds is demonstrated in Fig. 6. The 3D-printed scaffolds showed appropriate layer fidelity, according to the CAD, as shown in Fig. 2. The homogeneous layer fidelity was attributed to the suitable printability of the 30 : 70 (PVA : AA) blend and the rheological behavior of the PVA/AA/Ginger blend, as discussed in Sections 3.1 and 3.2. Moreover, the 3D-printed scaffolds demonstrated rough surfaces, which are required in skin tissue engineering applications, as fibroblasts adhere to the relatively rough surface. The scaffolds showed internal microporosity, as shown in Fig. 6(B). The internal porous structure of the scaffolds was attributed to the successful crosslinking of the PVA/AA/Ginger blend with CaCl_2_ and the freeze-drying process, which allowed the water to evaporate and created the porous sites. The internal porous structure may be responsible for the swelling of the scaffolds, which is beneficial for the migration and proliferation of the fibroblasts. Moreover, microporosities supported the adhesion of the fibroblasts to the surface of the scaffolds and promoted the growth of the fibroblasts, as discussed in the cell viability section. The mechanism of fibroblast cell adherence in the internal porous structure is illustrated in Fig. 6(C).

In order to quantitatively analyze the structural morphology of the fabricated PVA/AA/Ginger scaffolds, the pore size was calculated using ImageJ software. The histogram analysis of this quantitative study demonstrated a high degree of pore connectivity and size distribution, with pore sizes varying from 0.10 to 0.18 mm (100 to 180 micrometers) and with a peak frequency ranging from 0.14 to 0.16 mm.^41^ Such an ideal pore-size distribution and interconnectedness play a critical role in determining the functionality of the scaffolds in tissue-engineering applications. Fig. 6(A, B and E–J) shows that such an interconnected structure serves as an excellent environment for the deep penetration of fibroblast cells and their strong adhesion to the inner structure. Moreover, this quantitative pore-size distribution provided sufficient oxygen supply, nutrient exchange, and drug diffusion within the scaffold structure, which were vital in accelerating tissue formation and healing processes.^42^ Gillani *et al.*^21^ fabricated 3D-printed sodium alginate/AA/clove scaffolds that demonstrated internal porous structures due to crosslinking with CaCl_2_, with their morphology comparable to that of the 3D-printed PVA/AA/Ginger scaffolds developed in the present study. Batul *et al.*^29^ developed polyethylene oxide/guar gum-based 3D-printed hydrogels that showed favorable cellular viability with fibroblasts due to the microporous structure of the scaffolds, and the scaffold morphology was comparable to that observed in this study.

The SEM analyses confirmed the internal morphology of the scaffold. At a low magnification (Fig. 6(E)), the scaffold appeared as a porous, three-dimensional network that ran through the internal structure of the scaffold, thereby suggesting that freeze–drying efficiently maintained the interconnected morphology of the scaffold after CaCl_2_ cross-linking.^43^ SEM images at high magnifications (Fig. 6(F–H)) illustrated rough walls made up of the interconnected layers of polymers and fibrillar structures, as opposed to smooth, compact walls. This feature is important in that surface roughness provides a relatively large surface area for protein adsorption and also aids in cell–material interactions, thereby aiding cell attachment (Fig. 6).^44^

In addition, the SEM images at high magnifications (Fig. 6(I and J)) illustrated several fine particulate features distributed all over the polymer matrix. These particles reflected the ginger-extract molecules, which were entrapped in the PVA/AA cross-linked scaffold without showing signs of aggregation, thereby proving the efficient encapsulation and uniform distribution of the ginger extract within the scaffold matrix. There was no indication of any phase separation or structural degradation, thereby proving compatibility between PVA, agar-agar, and ginger extract used in the scaffold synthesis process (Fig. 4).

### 3.4. FTIR analysis

**Fig. 7:**
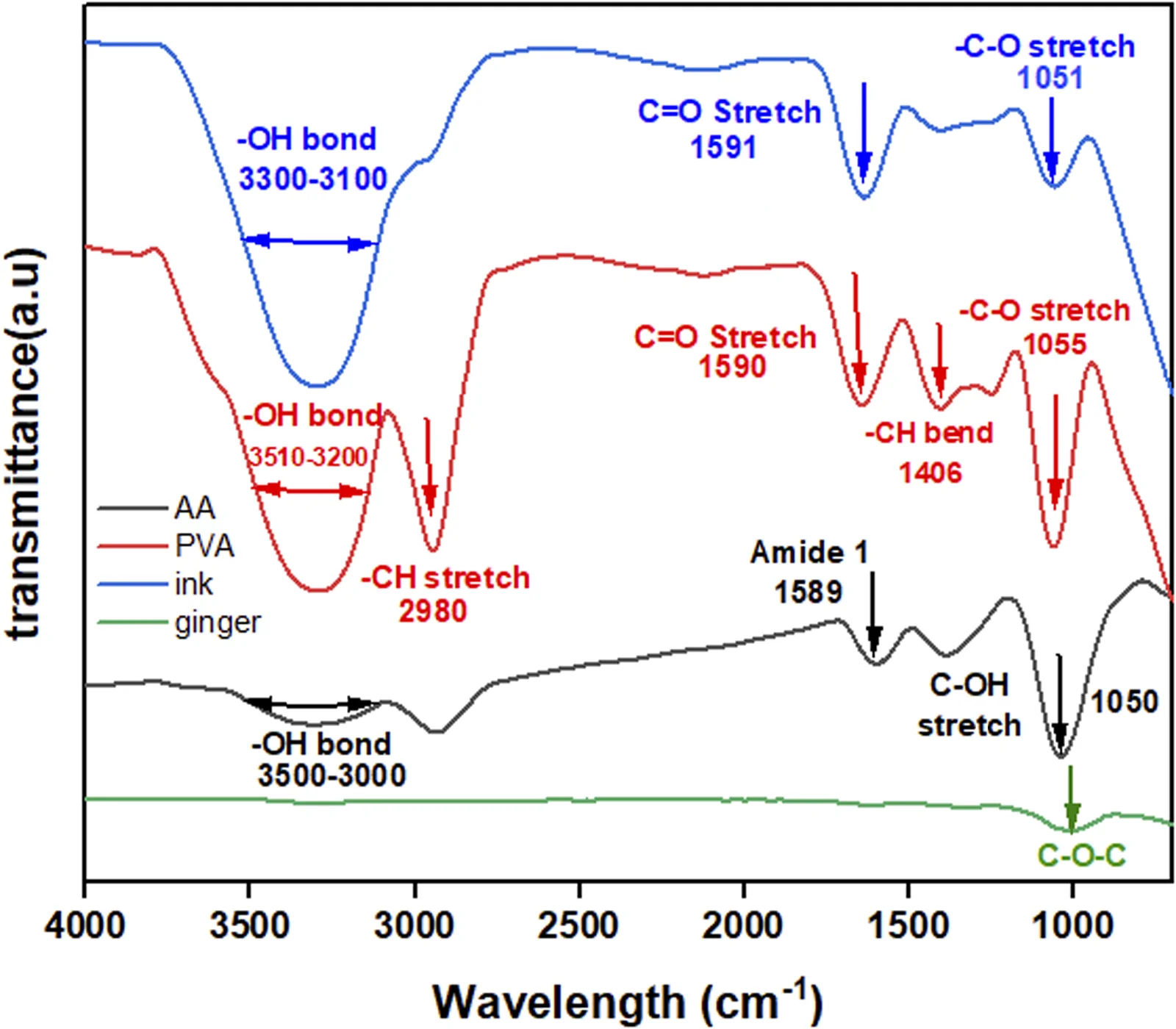
FTIR spectra of PVA, AA, and ginger and the crosslinked PVA/AA/Ginger scaffold.

The FTIR spectra of the individual materials (PVA, AA, and Ginger), along with the crosslinked PVA/AA/Ginger scaffold, are illustrated in Fig. 7. The broad spectra of 3510–3200 cm^−1^ was observed for PVA, which was due to the –OH stretching in PVA. The intense peak at 2980 cm^−1^ in PVA was due to the –CH stretching, followed by the minor peaks at 1590 cm^−1^ and 1406 cm^−1^ owing to C=O stretching and –CH bending, respectively. The intense peak at 1055 cm^−1^ was attributed to the –C–O stretching in PVA.^45–47^ The broad peak at 3500–3000 cm^−1^ was due to the –OH stretching in AA. The slight peak at 2960 cm^−1^ was attributed to the –CH stretching in AA. The minor peaks at 1589 cm^−1^ and 1416 cm^−1^ were due to the amide I and II stretching, followed by the intense peak at 1050 cm^−1^ due to the –C–OH vibrations.^21,48^ Ginger showed the FTIR peak at 1050 cm^−1^, which was attributed to the secondary alcoholic and ether linkages present in gingerol. An increase in the peak intensity of the –OH stretching at 3300–3100 cm^−1^ was observed for the crosslinked PVA/AA/Ginger scaffold, which was due to possible physical association between the polymer chains. The decrease in the peak intensity of C=O stretching and amide I stretching in AA at 1591 cm^−1^ indicated the ionic interactions between Ca^2+^ and the hydroxyl-rich polymer chains. The incorporation of ginger was inferred from the altered FTIR profile of the PVA/AA/Ginger scaffolds, particularly the changes in the –OH and fingerprint regions, while the 1051 cm^−1^ band represented overlapping C–O/C–OH contributions from PVA, AA, and ginger (Table 3). The notable differences in the peak shifts and intensities of individual materials and fabricated scaffolds showed molecular interactions between them. The –OH group stretching-vibration region confirmed intermolecular hydrogen bonds among the polymer molecules. The shift and diminution in the carboxylate vibrational range at 1600–1400 cm^−1^ indicated the ionic coordination with Ca^2+^ and, thus, the cross-linking reaction. The distinctive peaks for the functional groups of ginger, including secondary alcohols, were observed at 1000–1100 cm^−1^.^49^ The chemical reaction between PVA and AA using CaCl_2_ is given in eqn (5) (Fig. 5).5
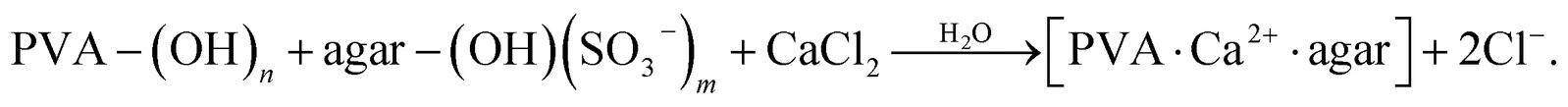


**Table 3:** FTIR labelling of the wavenumbers for the functional groups present in individual materials and the fabricated PVA/AA/Ginger scaffolds

Wavenumber (cm^−1^)	Functional group	Source component	Interpretation
*R*∼3200–3500	–OH stretching	PVA, AA	Hydrogen bonding; peak broadening indicates intermolecular interaction
∼2900	–CH stretching	PVA, AA	Polymer backbone confirmation
∼1600–1650	C=O/bound water	AA	Shift suggests ionic interaction with Ca^2+^
∼1400–1450	–CH bending/COO^−^ symmetric	AA	Involvement in Ca^2+^ coordination
∼1000–1100	C–O–C/C–OH	PVA, AA, and ginger	Overlapping peak; increased intensity indicates composite formation
∼1050	C–O/C–OH; oxygen-containing groups	PVA, AA, and ginger	Overlapping contribution; changes in the band profile support the incorporation of the ginger-containing phase

### 3.5. Mechanical study

**Fig. 8:**
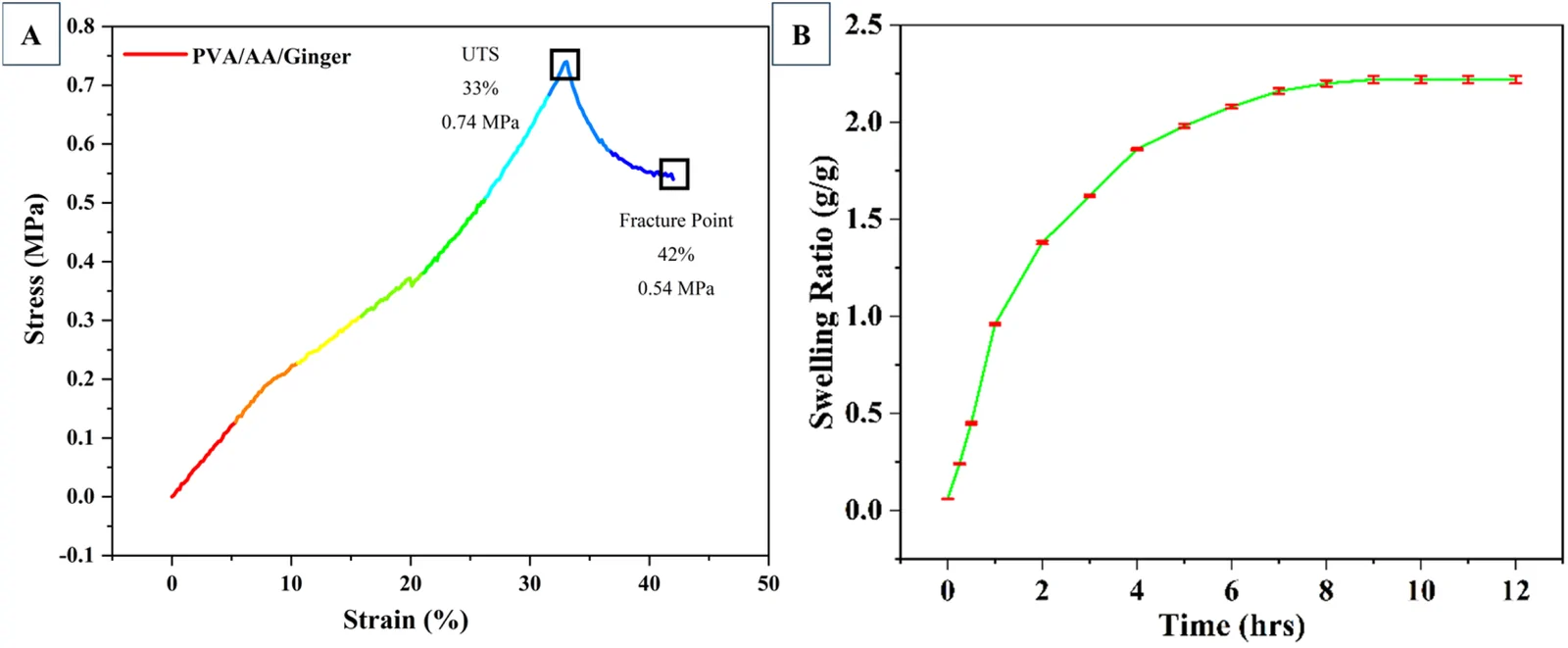
(A) Micro-tensile test conducted on the PVA/AA/Ginger scaffolds. (B) Swelling ratio (g g^−1^) of the 3D-printed PVA/AA/Ginger scaffolds after immersing in PBS for 12 h.

The tensile stress–strain properties of the fabricated scaffold demonstrated characteristic behavior for soft, hydrogel-based polymers. The stress–strain graph revealed a linear elastic region, with a smooth transition into nonlinear stress growth. Thus, ultimate tensile strength was achieved at 0.74 MPa, where strain was 33%; then, a gradual stress decrease began until the material broke under a strain value of 42% at 0.54 MPa, as shown in Fig. 8(A). Such behavior implied that the scaffold had undergone some structural changes after reaching its maximum stress point. The fracture occurred slowly rather than abruptly due to brittleness, which may be due to the crosslinked network. Such values of tensile strength are within the range reported for comparable polymeric wound-dressing systems.^50,51^ The PVA/AA/Ginger scaffolds are expected to be flexible rather than resistant to mechanical loads. Hence, for similar types of scaffolds made from PVA or polysaccharides, their tensile strength values were found to be in the range of 0.1–2 MPa, depending on the scaffold's composition. It was reported that the range of values for tensile strength for such systems as PVA-alginate and PVA-chitosan were in the interval of 0.3–1.5 MPa (Fig. 8A).^52^

In terms of wound healing, the trade-off between strength and flexibility outweighs the need for high mechanical strength. The natural skin has varying degrees of tensile strength, ranging from 2 to 30 MPa, yet engineered scaffolds do not necessarily have to attain all these values because the purpose of these materials is only temporary. Rather, they need enough elasticity that will enable movement and avoid irritation. The obtained elongation of around 42% and lack of catastrophic failure pointed out that the scaffold had ductility, which was beneficial in making conformable contact with irregular surface wounds.^53^ The mechanical properties could be attributed to the scaffold formulation and cross-linking strategy. The contribution of PVA was to provide chain entanglement and flexibility, while the agar offered a gel-like network. The formation of ionic cross-linking with Ca^2+^ involved reversible junctions to provide cohesion while still allowing deformability under applied stress. Bhardwaj *et al.*^54^ demonstrated that integrating hydrogel systems into wound dressings improved tensile strength and elongation, highlighting the importance of balanced mechanical properties for effective wound repair. Compared to such advanced bilayer systems, the present scaffold exhibited a comparable range of flexibility, although with a simpler composition and fabrication strategy. This moderate strength combined with high deformability is particularly advantageous for wound healing, as it allows the scaffold to conform to dynamic skin surfaces while maintaining structural integrity, which is critical for maintaining a moist environment and supporting tissue regeneration.

### 3.6. Swelling studies

The swelling behavior of the biomedical scaffolds plays a crucial role in skin tissue engineering applications, as it determines the scaffold's ability to absorb the biological fluids present in the human body. Scaffolds that exhibit swelling upon absorbing liquid are considered in wound healing applications, as the swelling provides a suitable environment for the fibroblasts to remain viable within the scaffolds.^55–57^ The swelling ratio (g g^−1^) of the 3D-printed PVA/AA/Ginger scaffolds is illustrated in Fig. 8(B). The 3D-printed scaffold showed an initial fast swelling until 2 h, with a swelling ratio of 22 and a swelling percentage of 2200%. The scaffold showed a sustained and controlled swelling ratio of ∼36 and a swelling percentage of 3566% until 12 h. The initial fast swelling ratio was due to the hydrophilic nature of PVA, AA and ginger. The hydrophilic functional groups present in PVA and AA, *i.e.* hydroxyl (–OH) and ether linkages, supported the adsorption of water on the surface of the scaffolds.^58,59^ Moreover, the interconnected porous structure of PVA/AA/Ginger, as shown in Fig. 6, facilitated the absorption of water on the porous sites of the 3D-printed hydrogels and resulted in improved swelling. The improved swelling ratio of the 3D-printed PVA/AA/Ginger scaffolds was expected to promote fibroblast cell migration and differentiation required in the wound healing process. The obtained swelling properties from the fabricated PVA/AA/Ginger scaffolds exhibited high hydrophilicity, which was essential for skin burn applications. Further, hydrophilicity is essential for nutrient exchange in cells, resulting in improved cell proliferation and protein attachment.^60^ Felfel *et al.*^61^ synthesized a hydrogel based on chitosan/agarose and showed a hydrophilicity of 1500% after 6 h. Zhu *et al.*^62^ developed PVA/cetyl-dimethylallyl ammonium chloride-based hydrogels, and the results of the swelling ratio were in comparison with those of the present study. Hussain *et al.*^63^ developed PVA/carboxymethyl cellulose-based hydrogels *via* the casting method, and the results of the swelling ratio were in comparison with those of the present study.

### 3.7. Degradation studies

**Fig. 9:**
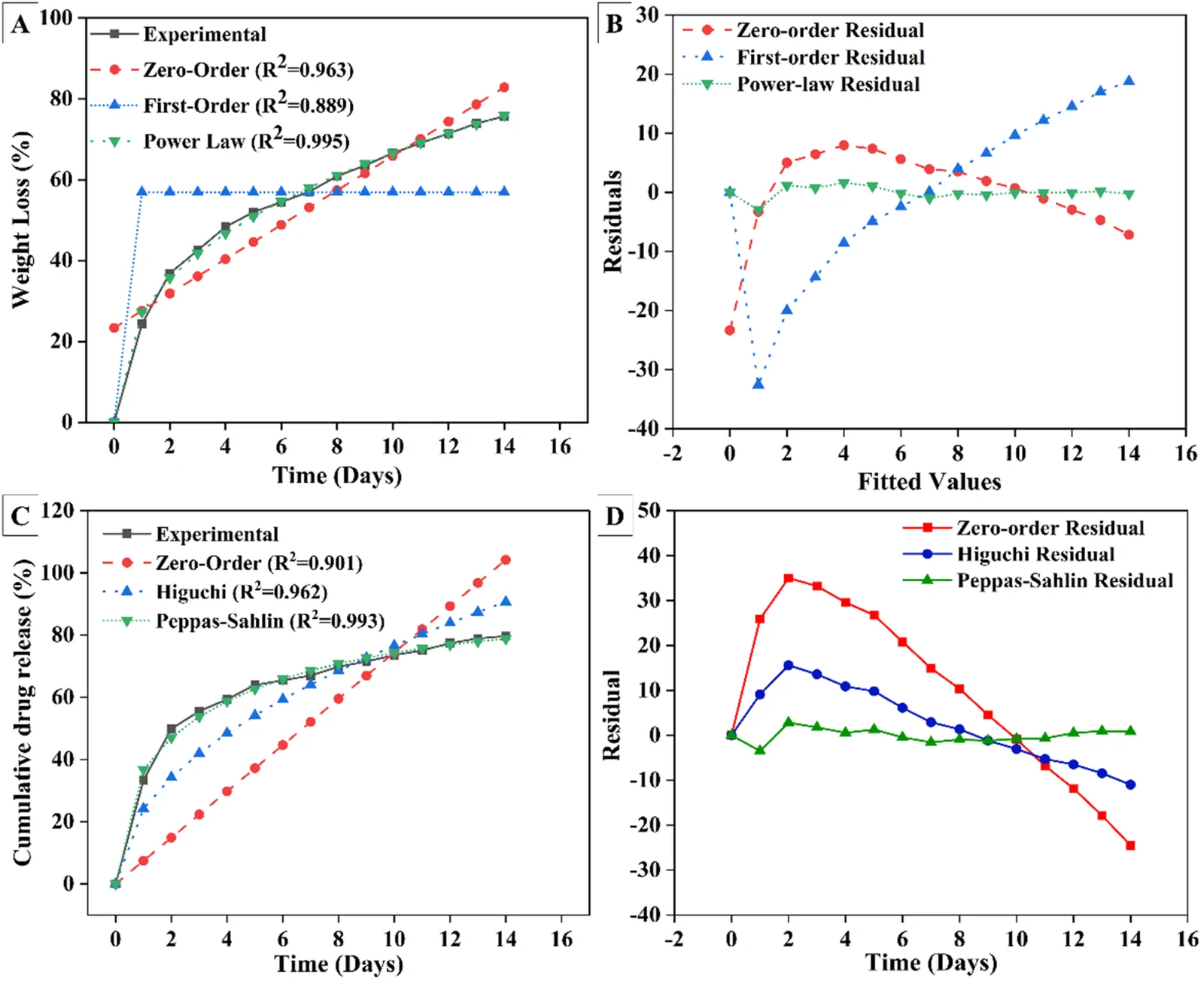
Weight loss (%) of the 3D-printed PVA/AA/Ginger scaffolds during the 14 days of immersion in PBS, with the *R*^2^ values of different mathematical models. (B) Residuals of the fitted degradation models. (C) Cumulative gingerol release from the 3D-printed PVA/AA/Ginger scaffolds during the 14 days of incubation, with the *R*^2^ values of different mathematical models. (D) Residuals of the fitted models.

The biodegradability of wound dressings is very important in skin tissue engineering applications, as it determines the ability of the dressings to degrade under certain biological conditions and release effective biological molecules to the wound site, providing synergistic biological effects. Biodegradable scaffolds promote the wound-healing process by degrading at the wound site and enhancing fibroblast migration and proliferation, which form the extracellular matrix (ECM) and result in skin regeneration.^64–66^ The weight loss (%) of the 3D-printed PVA/AA/Ginger scaffolds is illustrated in Fig. 9(A). The initial fast degradation of the scaffolds was observed until 2 days of immersion in PBS, with a weight loss (%) of ∼37%. The rapid degradation was due to the degree of crosslinking loss and the weak intermolecular bonding at the surface of the scaffolds, which allowed the molecular chains to break and degrade under simulated physiological conditions. The slow and sustained degradation was observed until day 14, with a weight loss (%) of ∼76%, which was attributed to the strong interconnection between PVA and AA, allowing the scaffolds to degrade slowly. Moreover, the degradable nature of PVA and AA and the degradable functional groups, *i.e.* hydroxyl, ester, glycosidic linkages, and carboxyl groups, allowed the scaffolds to degrade in PBS. The initial fast weight loss (%) provided the fast release of the bioactive ingredient, *i.e.* gingerol, which provided the antibacterial effect, as discussed in Section 3.7. In addition, different mathematical models were fitted to the degradation experimental data to observe the perfect fit for the degradation. The power law model proved to be the best fit for the degradation results, as the *R*^2^ for the power model was near 1, *i.e.* 0.995. The value of *R*^2^ for the zero-order model was 0.963, which was relatively lower than that of the power law model. The first-order model proved to be a poor fit for the degradation experimental values, as the value of *R*^2^ was far less than 1, *i.e.* 0.889. The residuals of the different models in comparison with the biodegradation values are demonstrated in Fig. 9(B). Mathematical models with residuals near 0 proved to be the best fit for the experimental values. The residuals of the first-order model were far from 0, which indicated that the model values were significantly different from the experimental values, proving the poor fit of the first-order model for the degradation experimental values. Improved fitting was observed for the zero-order model, with the values of residuals closer to 0 in comparison with the first-order model. However, the residual values were still away from 0, which proved to be a poor fit for the experimental values of degradation. The power law model proved to be the perfect fit for the experimental values of degradation, with the residuals close to 0. Hence, the power law model proved to be the perfect fit for the degradation of the 3D-printed PVA/AA/Ginger scaffolds. Gillani *et al.*^21^ developed 3D-printed sodium alginate/agar-agar-based hydrogels that showed suitable biodegradation, according to the requirements of skin regeneration applications, and the results of biodegradation were in comparison with those of the present study.

### 3.8. Drug-release kinetics

The release of the bioactive and antibacterial ingredients is very critical in skin regeneration applications, as the release of bioactive ingredients provides antibacterial effect and triggers specific markers that modulate the wound healing process. In the wound healing process, the initial burst release of antibacterial molecules is required within 48 h, as the pathogenic bacteria are expected to attack the wound site and hinder the healing process.^67–69^ The cumulative gingerol release from the 3D-printed PVA/AA/Ginger scaffolds is illustrated in Fig. 9(C). The scaffolds showed an initial gingerol burst release of ∼50% until 2 days of incubation, followed by the slow and sustained release of ∼80% until 14 days of incubation. The initial burst release of gingerol from the 3D-printed PVA/AA/Ginger scaffolds was attributed to the fast degradation of scaffolds until 2 days of incubation, as discussed in 3.6. Moreover, the porous sites of the scaffolds allowed the gingerol to be released from the surface of the scaffolds. The sustained release of gingerol from the scaffolds until day 14 was due to the strong crosslinking and strong interconnected matrix of PVA and AA, which allowed the gingerol to be released in a sustained and controlled manner. Moreover, the concentration gradient was also low in comparison to that on the first 2 days, which caused the gingerol to be released in a controlled way. The drug-loading efficiency showed the successful loading of ginger in the fabricated PVA/AA scaffold. According to the formulation, the theoretical loading of ginger was 125 µg per scaffold. However, through the UV-vis measurement of the dissolved scaffold, the actual loading of ginger was found to be 117 ± 3 µg per scaffold, resulting in a drug-loading efficiency of 93.6% ± 2.4% (*n* = 3). The high loading efficiency suggested that only a tiny portion of ginger was lost during printing, ionic crosslinking, and freeze-drying.^70^ The highly hydrophilic PVA/AA matrix entrapped the ginger molecules due to crosslinking in the polymeric matrix. The high loading efficiency supported the sustained drug release profile, where around 79% of ginger (around 92 µg) was released from the scaffold in 14 days, and the rest remained attached to the matrix.

Different mathematical models were fitted to the experimental values of gingerol release from the 3D-printed PVA/AA/Ginger scaffolds. The Peppas–Sahlin model proved to be the perfect fit for the gingerol-release experimental values, as the value of *R*^2^ for Peppas–Sahlin was 0.993, which was closer to 1 in comparison to both zero-order and Higuchi models. The residual analysis was carried out for the fitting values of mathematical models and the experimental values of gingerol release, as shown in Fig. 9(D). The values of residuals for the zero-order model were far from 0, which proved the poor fit of the model for the experimental values of biodegradation. In comparison to the zero-order model, the Higuchi model provided a better fit, with the residual values close to 0. In comparison to both zero-order and Higuchi models, the Peppas–Shalin model was the perfect fit for the experimental values of gingerol release from the 3D-printed PVA/AA/Ginger scaffolds, with the residual value close to 0. Hence, the Peppas–Sahlin model was the perfect fit for the release of gingerol from the 3D-printed PVA/AA/Ginger scaffolds. The initial burst release of the gingerol provided the antibacterial effect, as discussed in 3.8. The results of the cumulative release of gingerol from the scaffolds agreed with the biodegradation studies. Kazmi *et al.*^71^ developed PVA/carboxymethyl cellulose-based nanofibrous mats, and the mats showed an initial humic acid burst release, followed by a slow and sustained release, due to the crosslinked PVA/carboxymethyl cellulose matrix. The results of the study agreed with the results of this PVA/AA/Ginger scaffold study.

### 3.9. Antibacterial potential

**Fig. 10:**
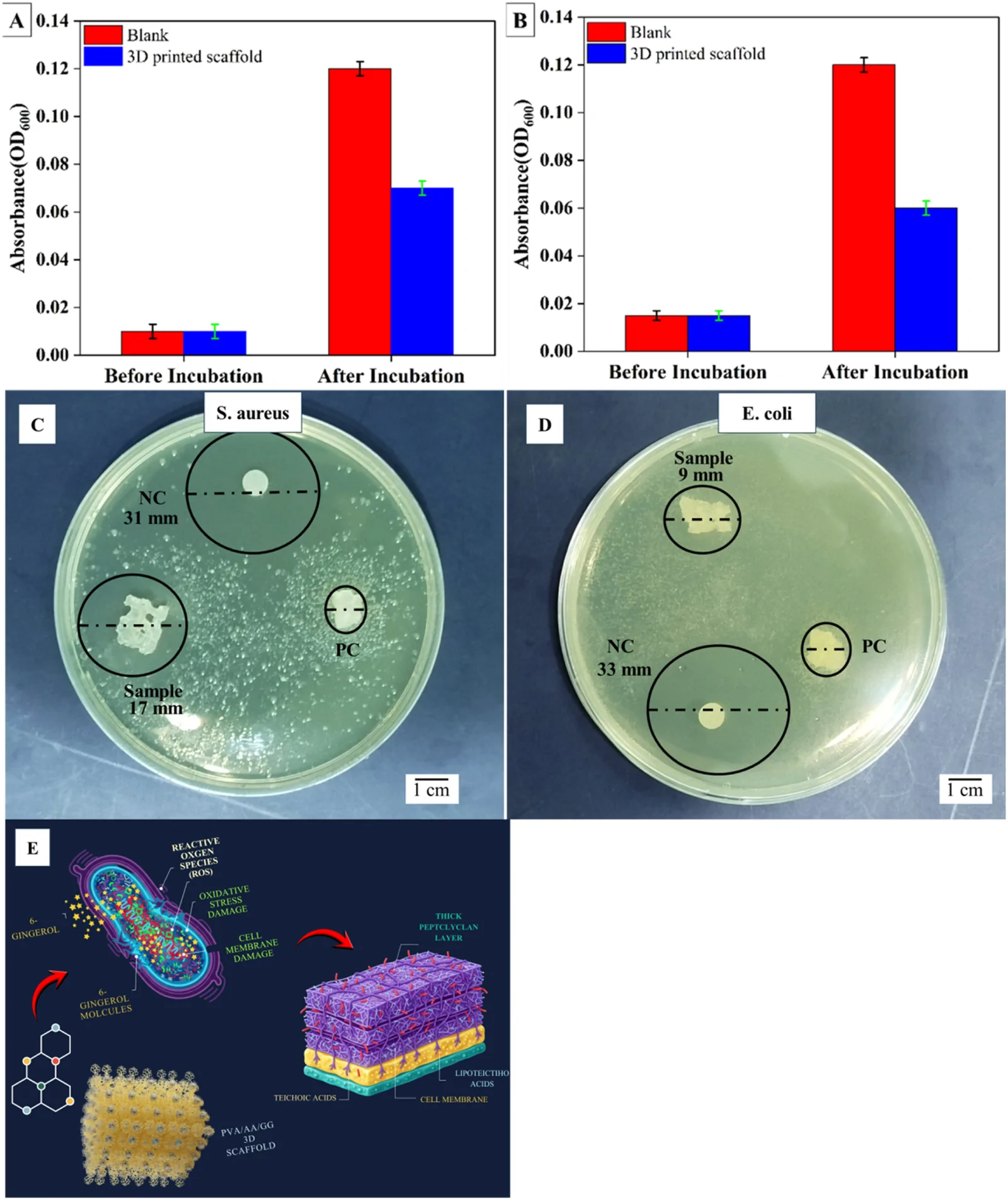
Antibacterial efficacy of the 3D-printed PVA/AA/Ginger scaffolds through turbidity against (A) *E. coli*. and (B) *S. aureus* and using the disk diffusion test against (C) *S. aureus* and (D) *E. coli*. (E) Mechanism of the antibacterial effect of gingerol (Designed using CanvaPro (Canva, 2026)).

The antibacterial behavior of wound dressings plays a crucial role in skin regeneration applications, as the attack of pathogenic bacteria on the wound site results in hindrance to the wound healing process and delayed wound healing. Wound dressings are expected to provide antibacterial effects during the first 48 h of burns.^72–74^ The antibacterial behavior of the 3D-printed PVA/AA/Ginger scaffolds is shown in Fig. 10. The scaffolds showed promising antibacterial effects against both pathogenic bacterial strains, *i.e. E. coli* and *S. aureus*. The antibacterial behavior of the scaffolds was due to the initial burst release of gingerol from the PVA/AA/Ginger scaffolds for up to 2 days, as discussed in Section 3.7. The lipophilic nature of gingerol may allow gingerol to enter the bacterial lipid bilayer, which increased the bacterial membrane permeability, causing the intracellular contents, *i.e.* potassium ions, proteins and nucleic acids, to leak from the bacterial membranes. The superior antibacterial behavior with *S. aureus* was due to its exposed peptidoglycan layer, which allowed the gingerol to damage the bacterial cell membrane. Moreover, gingerol had the potential to increase reactive oxygen species (ROS), which increases oxidative stress, resulting in the damage of the DNA, proteins and nucleic acids inside the bacterial cells. In addition, gingerol inhibited biofilm formation, as gingerol disrupted quorum sensing, which was majorly involved in bacterial cell–cell communication, resulting in enhanced antibacterial efficiency.^75–77^ The results of the antibacterial studies agreed with the drug-release kinetics of gingerol and degradation studies.

The antimicrobial efficacy of the fabricated scaffolds was tested by the agar diffusion method after an incubation period of 48 hours using *S. aureus* (Fig. 10(C)), a Gram-positive organism, and *E. coli* (Fig. 10(D)), a Gram-negative bacterium. The figures show that the control antibiotic (NC) displayed high antimicrobial activity, causing significant inhibition zones, with sizes of 31 mm for *S. aureus* and 33 mm for *E. coli*. This confirmed the effectiveness of the tests and the suitability of the tested bacterial cultures. Consistently, the control scaffold based on PVA/AA (PC) showed no antimicrobial effects, where bacterial growth extended right up to the boundary of the scaffold in both plates. This indicated the absence of antimicrobial activity in the control 3D matrix. However, the scaffold with ginger extract (Sample) showed inhibition zones around itself against both bacteria, with sizes of 17 mm for *S. aureus* and 9 mm for *E. coli*.^78,79^ This demonstrated that the active ingredients, such as gingerols, present in ginger were encapsulated in the scaffold and retained their bioactivity upon release into the environment. The clear difference in effectiveness between the two strains was due to the different structures of their bacterial cell walls.^80^ The *S. aureus* strain is a Gram-positive bacterium whose cell wall has a thick but porous peptidoglycan layer, which allows the easy penetration of phenolic components from plants into the cells, causing disruptions in the cell membrane and the leakage of cellular content. On the other hand, the *E. coli* strain is a Gram-negative bacterium that has a highly complex outer membrane called LPS. The outer membrane is known for being a strict barrier that inhibits the entrance of many hydrophobic and hydrophilic molecules.^81,82^ The inhibition zones in both samples were relatively smaller than those in the concentrated commercial antibiotics; it became clear that the inclusion of ginger in the matrix successfully inhibited bacterial growth. Thus, the developed biomaterial consisting of PVA, AA, and ginger is considered promising and can be used for various applications, especially when antimicrobial effects are required.

The relation of antibacterial activity with the release rate of ginger from the PVA/AA/Ginger composite within the first 48 h was also observed. Drug loading efficiency (described in Section 3.8) showed that the actual loading of ginger was 117.5 µg per scaffold, which corresponded to 94% loading efficiency. In the first 48 h, 50% of the loaded ginger was released, that is, 58.8 µg per scaffold. Such release rate corresponded to the antibacterial test, where 17 mm inhibition zones were observed against *S. aureus* and 9 mm against *E. coli*. The relatively small inhibition zone against *E. coli* may be due to the presence of an outer lipopolysaccharide membrane, which restricted phenolic compounds, such as gingerol, from reaching the cell wall of the bacteria. Thus, the antibacterial effect was directly associated with the release of approximately 59 µg of ginger during the burst phase, and the sustained release lasted 14 days.

### 3.10. Cell viability and VEGF release

**Fig. 11:**
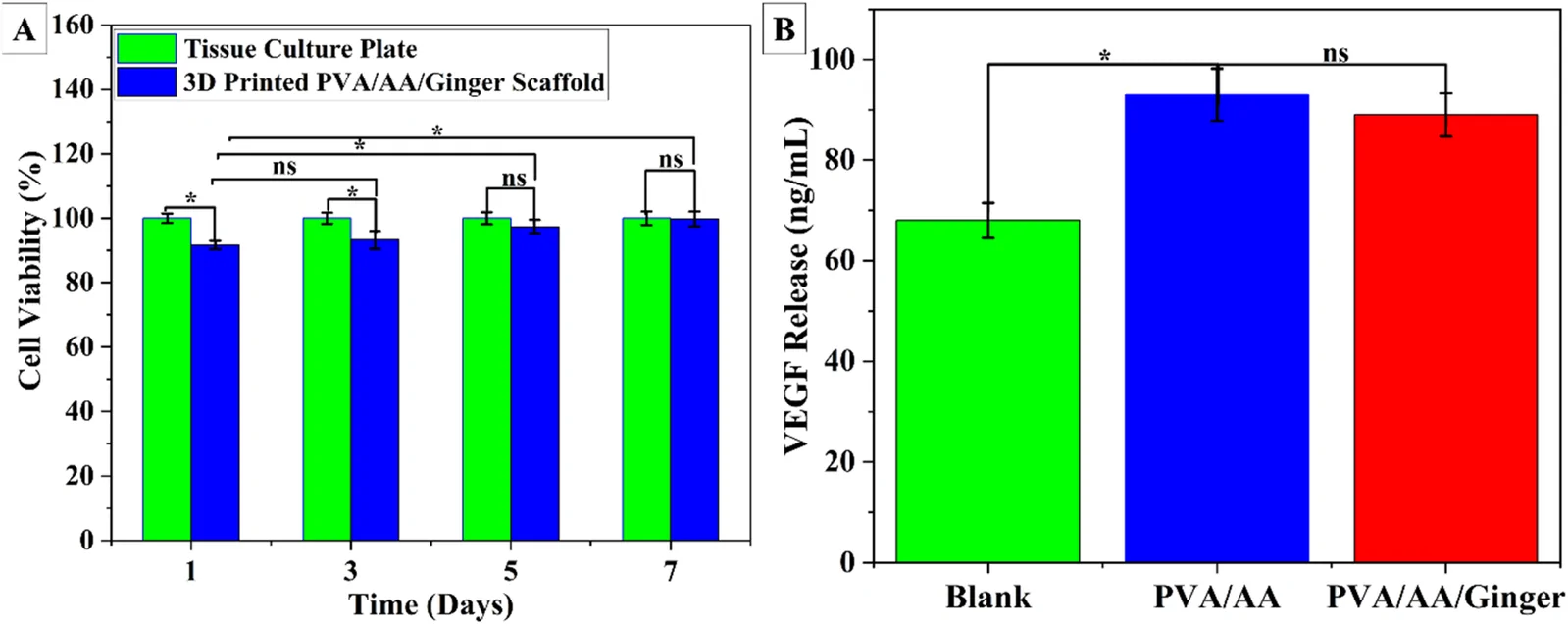
Biological investigations of the 3D-printed PVA/AA/Ginger scaffolds for (A) cell viability and (B) VEGF release. (One-way ANOVA was applied for the data analysis, with * indicating a significant difference and “ns” representing a non-significant difference (*p*  < 0.05) (*n* = 3)).

The cell viability of the 3D-printed PVA/AA/Ginger scaffolds with fibroblasts in comparison to a bare tissue culture plate (TCP) is demonstrated in Fig. 11(A). The 3D-printed scaffolds showed promising cell viabilities of 91.6%, 93.2%, 97.3%, and 99.7%, in comparison to the TCP, on days 1, 3, 5 and 7, respectively. This was due to the biocompatible materials, PVA and AA, which were viable for body contact. However, the slight decrease in viability was due to the release of ROS species from gingerol, which may slightly affect cell growth.^83–85^ The live/dead cells were checked through the trypan blue assay. The images of intact cells in the trypan blue assay are shown in the supplementary file. The figures show the visual evaluation of living cells based on the trypan blue exclusion test, in combination with the WST-8 cell viability test. The distinct, spherical structures with clear, unstained centers across both the green-filtered (A–C) and brightfield (D–F) views indicated intact cell membranes that successfully excluded the dye. The lack of dark, blue-stained entities confirmed a high level of cell viability and structural integrity under the tested conditions. This qualitative morphological observation effectively complemented the quantitative metabolic data typically generated by the WST-8 colorimetric assay.^86^

Moreover, the VEGF release was examined using an ELISA kit. The control PVA/AA scaffold exhibited higher VEGF release than the blank. However, the release of the PVA/AA/Ginger scaffolds was slightly lower than that of the control but higher than that of the blank. This may be due to the anti-inflammatory response of gingerol, which slightly suppressed the VEGF marker, as compared to the PVA/AA scaffold. In addition, being hydrophilic offered a porous cross-linked network, which helped in greater cellular attachment and proliferation. Moreover, the interaction of the bioactive compounds found in the scaffolds with integrin and tyrosine kinase receptors on the cellular surface initiated PI3K/Akt signaling pathways. This resulted in the activation of Akt through phosphorylation, resulting in an increase in the transcription of HIF-1 alpha, further resulting in an increase in VEGF release.^16,87,88^

### 3.11. *In Silico* analysis

**Fig. 12:**
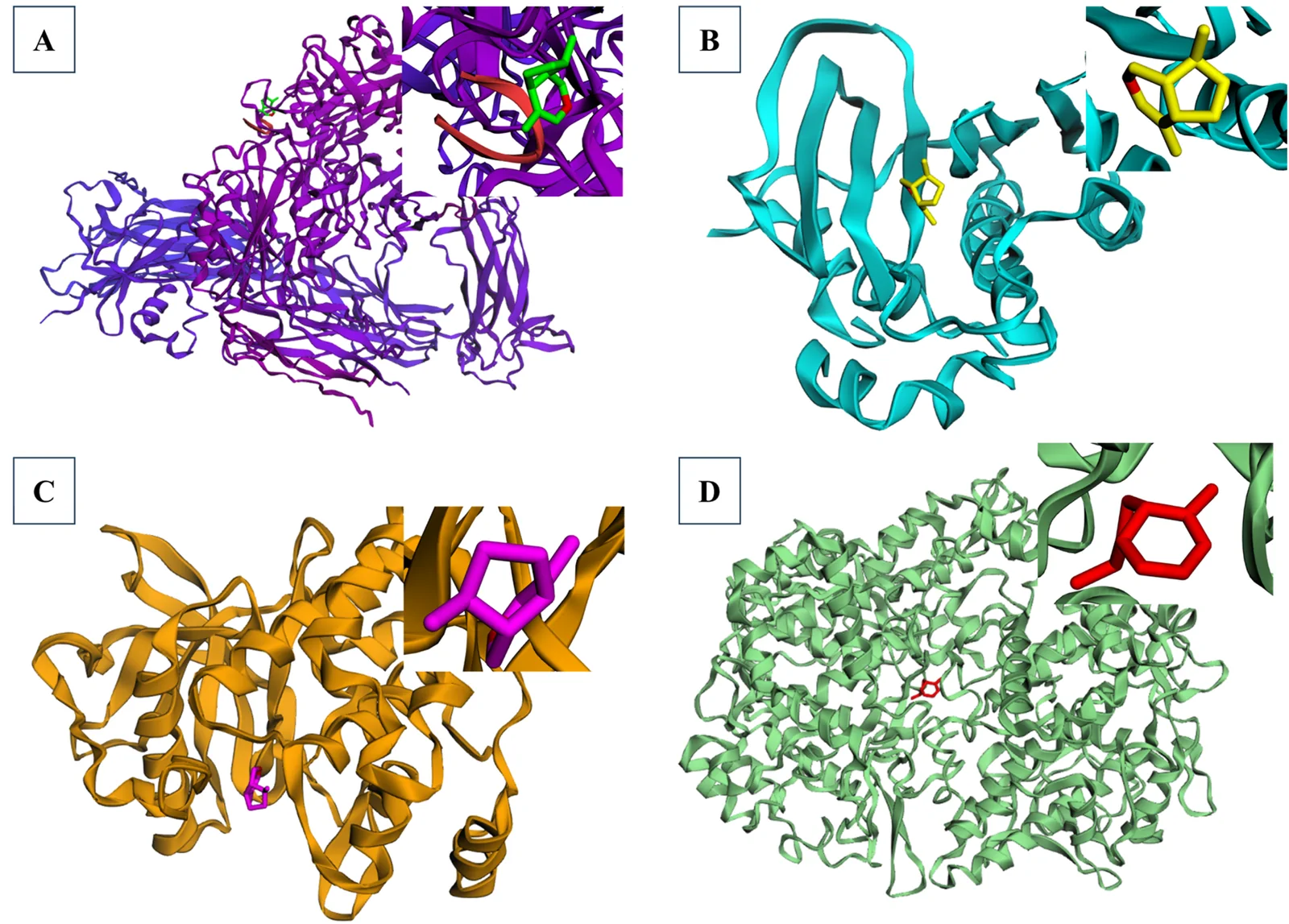
*In silico* molecular docking of 6-gingerol against targeted proteins. Panels presents docking simulations for: (A) integrin αvβ3 (purple), (B) DNA gyrase B (cyan), (C) VEGFR2 (orange), and (D) COX-2 (light green). Insets show zoomed in views detailing the “lock and key” binding interactions.

For a molecular validation of the multi-potent therapeutic utility of the 3D-printed PVA/AA/Ginger scaffolds, the *in silico* molecular docking study was carried out. [6]-Gingerol, which is the main active ingredient of ginger, was docked to four different biological targets that represented the different stages of wound healing: cell adhesion, antibacterial action (DNA gyrase), angiogenesis (VEGFR2) and anti-inflammatory action (COX-2). From the docking studies, [6]-gingerol demonstrated spontaneous binding activities for all four receptors, with negative binding affinities. The highest level of binding was observed for COX-2 (Panel D), with a binding energy of −6.036 kcal mol^−1^. From Fig. 12(D), it is evident that the ligand binds to the enzyme's hydrophobic catalytic pocket. This showed that the computational binding affinity of [6]-gingerol provided computational evidence for why the scaffold was bioactive, by controlling the inflammatory phase of the wound healing process through the inhibition of prostaglandin production.^89^

As far as tissue regeneration was concerned, the docking against integrin αvβ3 (Panel A) gave a binding affinity of −5.464 kcal mol^−1^. The depiction from Fig. 12(A) shows that the ligand bound to the interface between the alpha and beta subunits. This explained why the scaffolds presented in Fig. 11(A) showed high cell viability (99.7%) and high cell adhesion.^90,91^ In addition, the binding to VEGFR2 (Panel C), with an energy of −4.599 kcal mol^−1^, confirmed the prolonged VEGF secretion, as shown in Fig. 11(B). Docking (Fig. 12(C)) also suggested that gingerol may be able to bind the ATP-binding domain of the receptor and function as a signal regulator for angiogenesis in the proliferative phase of wound healing.^92^

Lastly, the antibacterial activity of PVA/AA/Ginger against *S. aureus* and *E. coli* (Fig. 10) was confirmed by docking on DNA gyrase B (Panel B), with a binding energy of −4.316 kcal mol^−1^. Though the docking energy was slightly lower than those of the other target sites, the successful docking into the bacterial enzymatic active site (Fig. 12(B)) suggested that gingerol interfered with bacteria replication, affording its antibacterial effect.^93^ In summary, the above *in silico* study revealed that the PVA/AA/Ginger scaffolds are not just physical structures but also biologically active systems, which interact with multiple target sites to speed up wound healing.

The binding affinities and molecular interactions are summarized in Table 4 and visualized in Fig. 12.

**Table 4:** Molecular docking binding affinities of [6]-gingerol against target proteins

Panel	Biological process	Target protein	PDB ID	Binding affinity (kcal mol^−1^)
A	Cell adhesion	Integrinαvβ3	1L5G	−5.464
B	Antibacterial	DNA gyrase B	6RQU	−4.316
C	Angiogenesis	VEGFR2	4ASE	−4.599
D	Anti-inflammation	COX-2	5KIR	−6.036

**Fig. 13:**
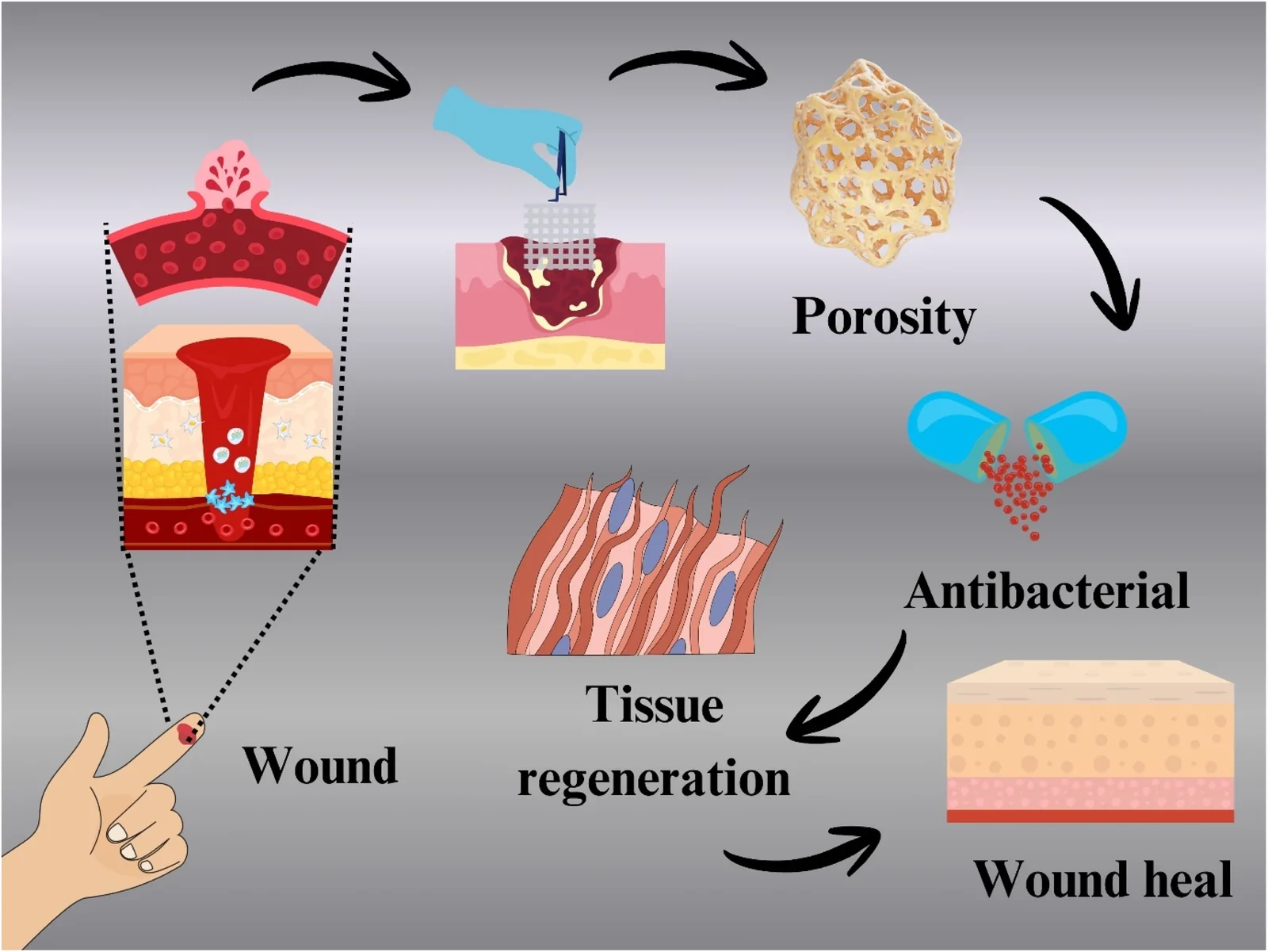
Schematic of the wound healing process of the PVA/AA/Ginger 3D scaffolds (Designed using CanvaPro (Canva, 2026)).

The engineered scaffold provided essential properties required for the soft tissue regeneration application. The biocompatible materials, PVA and AA, provided hydrophilicity through an inter-crosslinked porous structure. Moreover, the degradation of the 3D matrix released a bactericidal drug in a controlled manner, which helped in providing antimicrobial efficacy at the targeted wound site. Due to these effective properties, the wound healed, and the skin regenerated, as shown in the schematic presented in Fig. 13. Fig. 13 presents the schematic of the expected successive steps in the treatment of a skin wound using PVA/AA/Ginger scaffolds. Immediately after the injury, a grid-like mesh dressing was directly applied to the wounded tissue to support its recovery. The biocompatible scaffold exhibited very high porosity, allowing cell support, with the inclusion of an antibacterial property, depicted here by the drug-releasing capsule, to avoid infection. These act in conjunction to afford active tissue regeneration and include the ability of biological fibers and cells to rebuild quite effectively, leading to the complete healing of the wound and restored healthy and intact skin.

## 4. Conclusions

In this study, the primary 3D PVA/AA/Ginger network was fabricated *via* the DIW technique and investigated under *in vitro* conditions. The fabricated scaffolds exhibited a highly porous architecture, enabling efficient fluid uptake and supporting a moist environment suitable for wound healing applications. The ionically crosslinked network contributed to maintaining matrix stability under physiological conditions while allowing gradual disintegration over time. Additionally, the system provided controlled antibacterial efficacy with the degradation of the matrix. Cytocompatibility assessment through metabolic activity analysis confirmed that the scaffold provided a favorable environment for cell viability. Furthermore, the scaffold demonstrated cytocompatibility and characteristics that supported its potential use in regenerative wound dressing applications. As the current study is limited to the *in vitro* studies, *in vivo* and clinical studies will explore more on these fabricated 3D-printed PVA/AA/Ginger scaffolds.

## Author contributions

Rahila Batul and Rimsha Areej: conceptualization, methodology, writing – original draft, visualization, investigation. Muhammad Sameet Ismat, Syed Muneeb Haider Gillani, and Abdul Khaliq: writing – original draft, methodology, data curation. Weaam Mohamed Khoj Ali, Hanan Abdel Mawgoud Atia Soliman, and Noreen Akhtar: methodology, writing – original draft. Muhammad Atiq Ur Rehman: writing – reviewing and editing, funding acquisition, project administration, investigation, visualization.

## Conflicts of interest

There are no conflicts to declare.

## Data Availability

The data that support the findings of this study are available from the corresponding author upon request.
